# Systems biology and network pharmacology of frailty reveal novel epigenetic targets and mechanisms

**DOI:** 10.1038/s41598-019-47087-7

**Published:** 2019-07-22

**Authors:** J. C. Gomez-Verjan, R. Ramírez-Aldana, M. U. Pérez-Zepeda, R. Quiroz-Baez, A. Luna-López, L. M. Gutierrez Robledo

**Affiliations:** 1 Instituto Nacional de Geriatría (INGER), Mexico City, Mexico; 2 Geriatric Medicine Research, Dalhousie University and Nova Scotia Health Authority, Halifax, NS Canada

**Keywords:** Systems biology, Epigenetics

## Abstract

Frailty is an age-associated condition, characterized by an inappropriate response to stress that results in a higher frequency of adverse outcomes (e.g., mortality, institutionalization and disability). Some light has been shed over its genetic background, but this is still a matter of debate. In the present study, we used network biology to analyze the interactome of frailty-related genes at different levels to relate them with pathways, clinical deficits and drugs with potential therapeutic implications. Significant pathways involved in frailty: apoptosis, proteolysis, muscle proliferation, and inflammation; genes as *FN1, APP, CREBBP, EGFR* playing a role as hubs and bottlenecks in the interactome network and epigenetic factors as *HIST1H3* cluster and *miR200* family were also involved. When connecting clinical deficits and genes, we identified five clusters that give insights into the biology of frailty: cancer, glucocorticoid receptor, TNF-α, myostatin, angiotensin converter enzyme, ApoE, interleukine-12 and −18. Finally, when performing network pharmacology analysis of the target nodes, some compounds were identified as potentially therapeutic (e.g., epigallocatechin gallate and antirheumatic agents); while some other substances appeared to be toxicants that may be involved in the development of this condition.

## Introduction

As we age, physiological functions deteriorate, and these changes lead our organism to become vulnerable and to express age-associated disorders^1^. Among them, frailty, an age-associated condition resulting in an inappropriate response to stress and a higher frequency of adverse outcomes including, mortality, institutionalization and disability^2^. This condition results from the complex nature of *Homo sapiens* and a lifetime of environmental influences^2^. Light has been shed over the possible genetic factors involved in the origins of this condition; however, to what extent the heritability and genetic background affect an individual’s frailty status is still a matter of debate, mainly because of the multifactorial nature of this condition^3^. For instance, Dato *et al*. found that the genetic component was higher in men than in females^4^; nevertheless, the genetic background is not always the same and depends on several genes associated with the organism’s homeodynamic mechanisms as well as with an environmental relationship yet unknown.

Novel approaches are needed in order to disentangle the different characteristics of frailty and to narrow the gap in knowledge about this condition with a systemic view. The latest technological advances in genomics allow to use different approaches to analyze complete biological systems at different levels of complexity. In this context, systems biology, often referred as network biology^5^, has emerged over the last years as a novel paradigm in the field of big data biology aiming to improve our understanding of system-level molecular interactions (interactomics) underlying complex cellular processes, which lead to organ dysfunction; and eventually to clinical expression. These phenomena can be better analyzed using biological networks due to the fact that they possess several elements interacting with each other at different levels and emerging properties^6^. Biological networks; represented as molecular diagrams of the cell and its functions, enable to visualize several molecular interactions. Moreover, network analysis allows us to advance into the discovery of novel potential relationships and previously undiscovered paths through non-related molecules, enabling to figure the complete framework of multilevel cellular functions^6^. Network analysis is the newest available approach to the analysis of the system as a whole, aiming to elucidate the molecular mechanisms of complex age-related diseases and conditions such as frailty^7^.

Network pharmacology is another application tool in systems biology, aiming to mitigate the time-consuming process of drug discovery^8^. It helps to understand drug interactions with different targets and diseases from a network point of view^9^. These approaches, combined with other chemoinformatic methods synergize the drug repurposing process, since they allow to discover the therapeutic potential of already known compounds with novel indications^10^. These approaches are often used to discover new mechanisms of action and possible adverse effects of drugs^8^. Several research groups have used network pharmacology for different application, for instance, to explore bioactive ethnobotanical compounds with known use and to repurpose them for other pharmacological use^11,12^. Other example is metformin that has been repurposed as a chemo-preventive drug and is already on clinical trials^13^. So, these methods are useful to help reposition already used drugs (which have already proved to be safe in clinical trials) and to reveal possible novel mechanisms of action.

In the present study, we contribute both: to improve the understanding of the biology of frailty using different network biology analysis, and in therapeutics through network pharmacology analysis discovering novel possible targets and compounds (drugs and toxicants) that could be useful in this condition.

## Results

**Figure 1:**
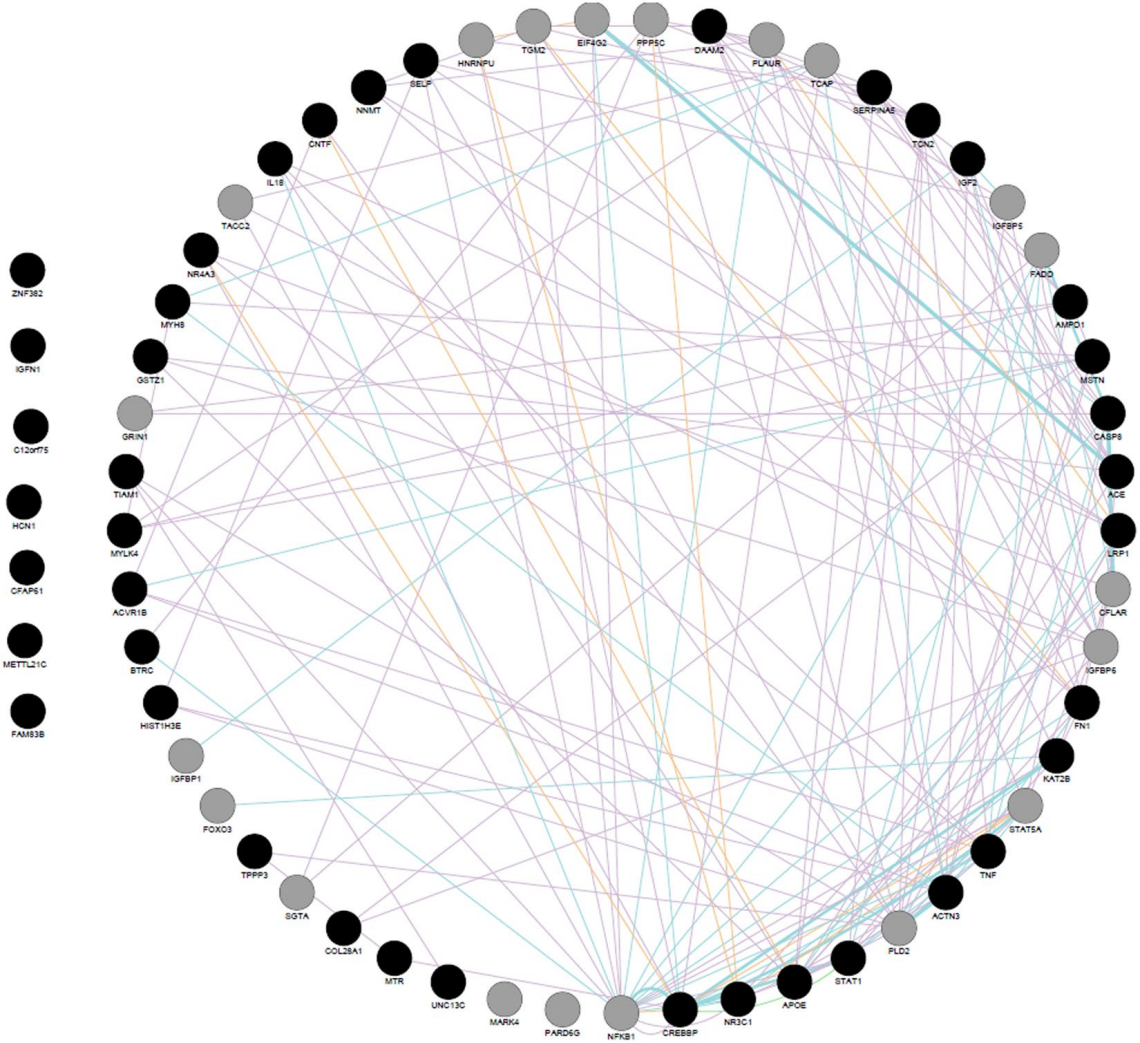
Network biology of the interactions among the genes highly related to frailty. To build the network, we use frailty-related genes along with novel genes obtained by GeneMANIA^38^ prediction server. Central genes (black) highly related genes (grey). Orange lines show physical interactions. Blue lines show interactions shared by common signaling pathways. Purple lines indicate co-expression. Isolated nodes are genes with no interaction.

In Fig. 1, is presented the network image showing the frailty-related genes^14,15^ (Supplementary Table 1S), along with highly related genes according to different types of interactions (physical interactions, shared common signaling pathways, and co-expression information of frailty-related genes).Through enrichment analysis of such genes, using different databases of pathways and diseases (Table 1), we observed that the most enriched pathways were apoptosis, proteolysis, smooth muscle proliferation, cytokines, interleukins signaling and the inflammatory process that happened to be the most related pathway. Interestingly, nervous system diseases and proteostasis deficiencies seem to be also highly related (Table 1).

**Table 1:** Pathway and disease enrichment analysis of genes resulted from interactome analysis of frailty-related genes accordingly to CTD, GEASPY and DAVID databases.

Pathway Enrichment Analysis	Pathway ID	Corrected P-value	Annotated Genes	DB
Regulation of the apoptotic process	GO:0042981	1.04E-4	13	DAVID
Positive regulation of proteolysis	GO: 0045862	1.91E-4	6	GSEAPY
Regulation of smooth muscle proliferation	KEGG	1.26E-4	6	GSEAPY
Death-inducing signalling complex assembly	GO: 0071550	2.62E-5	8	GSEAPY
Cytokine signalling in the immune system	REACT: HSA-1280215	3.09E-5	9	CTD
Signalling by Interleukins	REACT: HSA-449147	2.88E-5	8	CTD
Cellular response to organic cyclic compounds	GO:0071407	4.5E-5	5	DAVID
**Disease Enrichment Analysis**	**Disease ID**	**Corrected P-value**	**Annotated Genes Quantity**	**DB**
Nervous System Disease	CNS-diseases MESH: D009422	1.53E-6	16	CTD
Hypertrophy	Pathology (anatomical condition) MESH: D006984	1.11E-5	6	CTD
Cardiovascular disease	Cardiovascular diseases MESH: D002318	3.36E-5	11	CTD
Proteostasis deficiencies	Metabolic diseases MESH: D057165	6.40E-5	5	CTD
Neoplasms	Cancer MESH: D009369	7.75E-5	16	CTD
Brain diseases	Nervous system disease MESH: D001927	1.15E-4	10	CTD
Autoimmune diseases	Immune system disease MESH: D001327	6.10E-4	7	CTD
Urologic diseases	Urogenital disease MESH: D014570	7.9E-4	8	CTD

The table highlights the most significantly enriched pathways and diseases chosen accordingly to p-value < 0.05 corrected by the Benjamini-Hochberg procedure and separated accordingly with Pathways and Diseases. Frailty-related genes, along with novel genes obtained by GeneMANIA^38^ were used.

**Figure 2:**
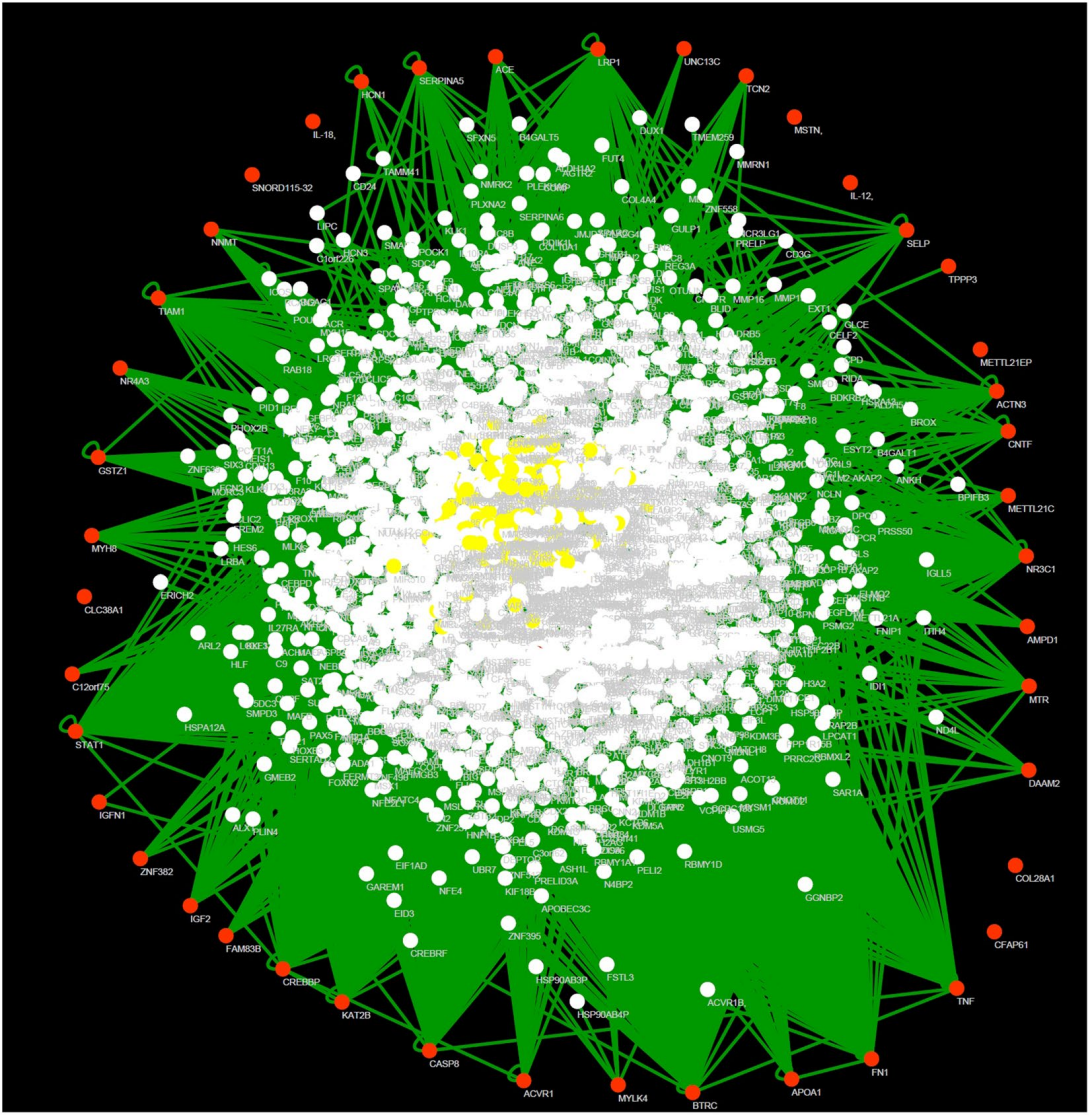
Network biology for protein-protein interactions (PPI) and miRNA-Protein interactions constructed with frailty-related genes as seeds. To build the network, we used BisoGenet^40^ plugin for Cytoscape (v. 3.7). Nodes represent proteins and miRNAs. Orange nodes represent those frailty-related genes (seeds). White nodes represent the first neighbor’s protein of the seeds, and yellow nodes represent miRNAs. Edges (green) represent PPI and miRNA-protein interactions.

In Fig. 2, is presented the interactome network corresponding to the protein-protein interactions (PPI) as well as protein-miRNA interactions with frailty-genes^14,15^. Network analysis identifies the more critical hubs and bottlenecks in the network (Table 2). Remarkably, there are transcription factors and proteins as Histone cluster 1 H3 family member H as hubs and bottlenecks. The second section of Table 2 shows the miRNAs hubs involved in the network and disorders related with them, where mir200 is among the most connected family of miRNAs (the complete list of miRNAs is in Supplementary Table 2S).

**Table 2:** Top 10 hubs and bottlenecks of proteins and miRNAs obtained from BisoGenet^40^ network.

Top Hubs		Top Bottlenecks	
Name	Functions/Related pathway	Name	Functions
FN1	Fibronectin-1/ERK signaling pathway(involved in cell adhesion and motility, wound healing, essential for osteoblast mineralisation)	FN1	Fibronectin-1/ERK signalling pathway (involved in cell adhesion and motility, wound healing, essential for osteoblast mineralisation)
NTRK1	Neutrophic receptor tyrosine kinase 1/MAPK pathways (insensitivity in pain and Thyroid carcinoma)	EGFR	Epidermal Growth Factor Receptor/ERK signalling pathway (Cancer)
CUL3	Cullin-3/MHC-1 mediated pathway (associated with ubiquitin-protein transferase activity)	APP	Amyloid beta precursor protein/TLR4 signalling (Alzheimer Disease)
TP53	Tumour protein P53/cell cycle, apoptosis senescence, DNA repair (Cancer)	FBXW11	F-box protein and WD repeat domain containing 11/Cell cycle and mitosis
HIST1H3H	Histone cluster 1 H3 family member H/Cell cycle and mitosis (Glioma)	COMMD3-BMI1	COMM domain-containing protein 3 and polycomb complex protein BMI-1 gene/micro RNA and Cancer and C-YC
HIST1H3E	Histone cluster 1 H3 family member H/Cell cycle and mitosis (Glioma)	CREBBP	CREB binding protein/DNA binding transcriptional factor
MCM2	Minichromosome Maintenance complex component 2/Cell cycle and DNA replication (Deafness)	CAND1	Cullin associated, and neddylation dissociated 1/proteasome system (Hypertension)
HIST1H3A	Histone cluster 1 H3 family member A/Cell cycle and mitosis (Glioma)	MIR367	MicroRNA −367/(Cancer)
APP	Amyloid beta precursor protein/TLR4 signalling (Alzheimer Disease)	HIST1H3E	Histone cluster 1 H3 family member H/Cell cycle and mitosis (Glioma)
HIST1H3J	Histone cluster 1 H3 family member J/Cell cycle and mitosis (Glioma)	EP300	E1A-associated cellular p300 transcriptional co-activator protein/Histone acetyltransferase (Cancer)
**miRNA**’**s associated with frailty with highest degrees**			
**miRNA**	**Degree **	**Diseases related to miRNA**	
MIR200B	147	Proangiogenic effect, Related with oral squamous cell carcinoma, colorectal cancer and melanoma	
MIR429	142	Regulates metastasis, activation of NF-kB and targets ECM Related with Ovarian, Lung Cancer and Coronary heart disease	
MIR130A	138	Regulates Hepatitis C and B virus replication promotes keratinocyte viability and migration, regulates STK40-mediated NF-kB pathway Related to Lung Cancer and Polymyositis	
MIR200C	138	Targets ECM and transcription factor PTEN Related with Renal cell carcinoma and Endometrial Cancer	
MIR130B	134	Targets ARHGAP-1, regulates proliferation and apoptosis of glioma cells Related with T-Cell Leukemia and cardiomyocyte hypertrophy	
MIR144	132	Targets ZFX and inhibits hepatocellular carcinoma cell and migration, regulates oxidative stress in SH-SY5Y cells Related with sickle cell disease and thalassemia	
MIR518E	131	Related with progressive supranuclear palsy	
MIR200A	130	Targets ECM and membrane receptors Related with meningioma, hepatocellular carcinoma, and ovarian cancer	
MIR29C	129	Regulates LOXL2 gene, targets CPEB4 and inhibits MAPK pathway Related with Huntington disease, renal cell carcinoma, cardiovascular disease, Rhabdomyosarcoma and metastatic brain tumour	
MIR518F	129	No disorder related	

The table indicates the hubs and bottlenecks detected using the cytoHubba plugin by Cytoscape^41^ and the top miRNAs associated with frailty.

**Figure 3:**
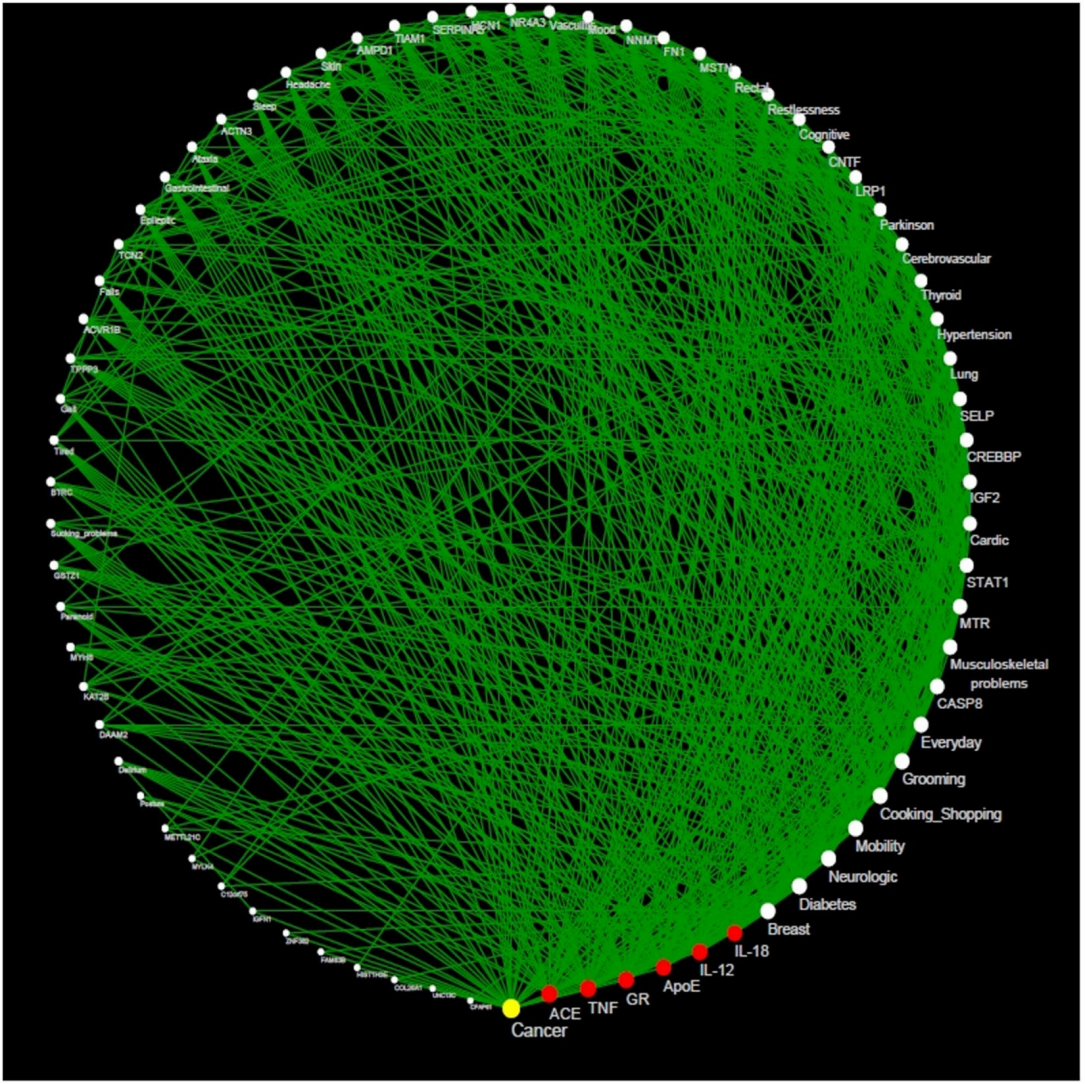
Network connecting Genes and Frailty Deficits. Nodes are deficits and genes. To build the network, we used information from weighted sociomatrix and used Cytoscape (v. 3.7.1) to draw it. Edges (green) are original experimental papers where genes correlate with deficits *in vivo*; *in vitro* or clinical data (Reviews and original papers with non-human models were excluded). Node size represents the degree. Yellow nodes are the most connected, red nodes are the next five most connected, and white nodes the other genes or deficits.

**Figure 4:**
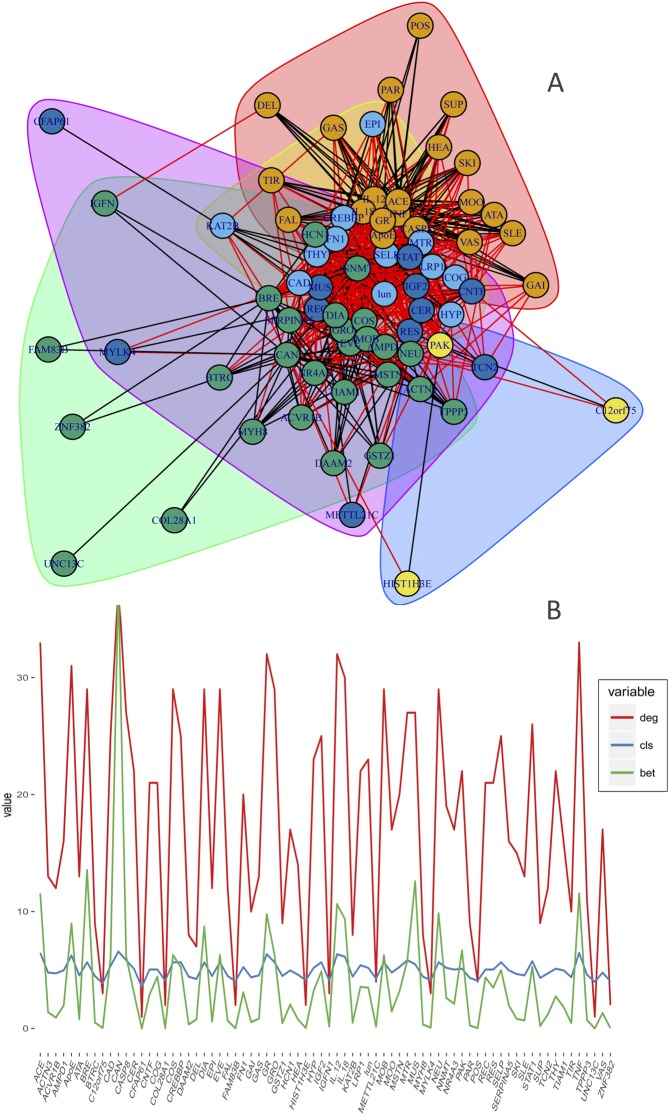
Clustering analysis of genes and deficits. Clustering analysis: (**A**) Network clustering associated with a network whose nodes are deficits and genes. (**B**) Centrality measures; degree (red), closeness (blue), and betweenness (green), associated with each node in the genes and deficits network.

Since the frailty index has been reported to be a clinically useful construct, integrated by a number of deficits from multiple body systems, we used it, in order to relate such deficits (Supplementary Table 3S) with potential frailty-related genes (Supplementary Dataset 1). Figure 3 shows the network topology of such relation, ordered counterclockwise from less to more related nodes, interestingly, cancer and five target genes (IL-8, IL-12, ACE, GR, ApoE, TNF) are the most connected nodes in the network. Besides, in search of new associations and potential mechanisms of interaction, we performed a clustering analysis (see methodology section) since we were searching for new associations and mechanisms. Figure 4 and Supplementary Table 4S, present results for such clustering analyses, as well as the behavior of the primary network properties, in which we identified 5 clusters of deficits and genes.

**Figure 5:**
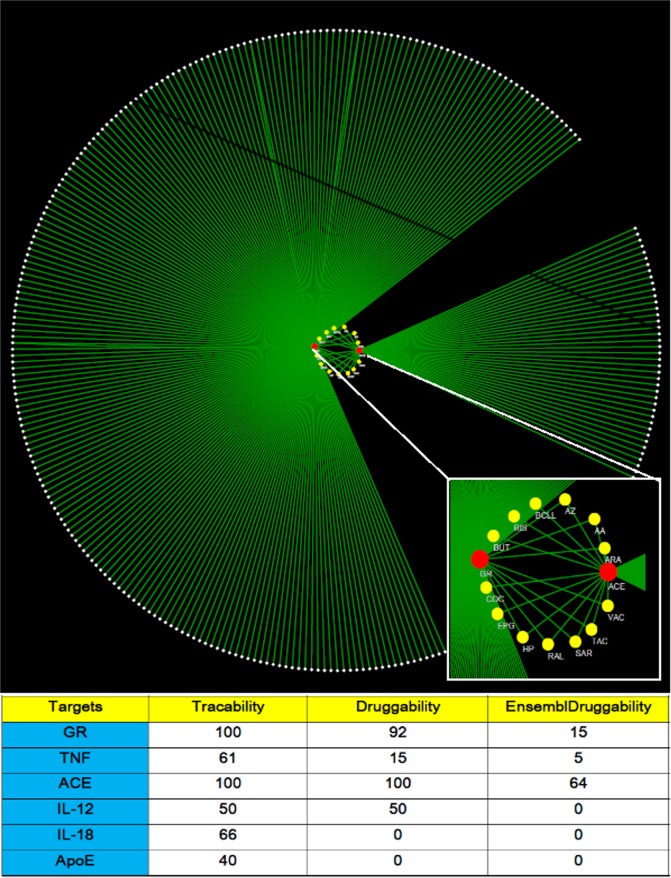
Network of ACE and GR and associated chemical molecules. The network was built with Cytoscape (v. 3.7.1) from data obtained from CTD for the most druggable targets ACE and GR. White nodes represent compounds interacting only with one target, yellow nodes represent compounds (drugs and toxicants) interacting with both targets, and red nodes represent the targets. Edges (green) are different types of interactions of compounds with targets accordingly to CTD. The table indicates the druggability parameters for each of the five possible targets.

In order to advance in the proposal of possible therapeutic alternatives, from our results, we explored the druggability values of the potential targets, identified because of their connectedness in the network (Figs 3, 4). As presented in Fig. 5, GR and ACE were the targets with more potential, according to their values of druggability and tractability. Therefore, in the next step, we used such targets in a network pharmacology analysis to find any possible related drug molecules. Results on Table 3 and Supplementary Table 5S show different molecules that interact with both GR and ACE. Interestingly, antirheumatic agents (mainly small molecules) already used in clinical practice could modulate both receptors. Additionally, a set of toxic compounds may be showing potential interaction with these two targets.

**Table 3:** Compounds targeting GR and ACE.

Compound	Characteristic
Ascorbic acid	Antioxidant (Vitamin C)
Antirheumatic agents	Particularly, conventional disease-modifying antirheumatic drugs: cyclosporin, cyclophosphamide, hydroxychloroquine, leflunomide, methotrexate, mycophenolate and sulfasalazine
Valproic acid	Anticonvulsant drug
Tetradecanoylphorbol Acetate	Natural activators of classic PKC also used for the treatment of haematological cancer
Raloxifene	A drug used for osteoporosis and breast cancer
Epigallocatechin gallate	Antioxidant in green and black tea *Camelia sinensis*
Cocaine	Abuse drug
Butyrates	Derivatives of butyric acid that contain the carboxypropane structure correlate with anticancer and anti-inflammatory effects
Benzyloxy Carbonyl Leucyl-leucyl-leucine aldehyde	A molecule used as a proteasome inhibitor
Bisphenol A	Toxic
Sodium Arsenite	Toxic
Atrazine	Toxic
Hydrogen Peroxide	Toxic-Used as antiseptic

The table indicates both drugs and toxicants, which interact with the most druggable targets (ACE and GR), accordingly to the CTD database.

## Discussion

A better understanding of molecular pathways involved in frailty and its clinical outcomes may contribute to improving the likelihood of a healthy aging. However, there is still controversy about the molecular mechanisms and pathways involved in this complex process due to its multifactorial nature. Therefore, in the present study, to contribute to a better understanding of the complexity of the systems involved in frailty biology, we used network biology analysis. Different types of centrality measures were used to strengthen results. These results allowed additional analyses such as drug repurposing through network pharmacology, identifying possible candidates for the intervention of this condition.

The most enriched pathways of frailty-related genes (Table 1)^14,15^, have a strong association with homeostatic mechanisms which are known to be affected both in aging and frailty. For example, failure in apoptosis could lead to errors in several regulatory processes that remove damage and aberrant cells from the body; with the accumulative damage during aging an increased apoptotic activity increasing tissue damage. Besides, apoptotic activity increases proteolysis signaling, leading to tissue atrophy and a pro-inflammatory response^16^. A low-grade chronic pro-inflammatory response seems to contribute to several age-related pathologies including cancer, cardiovascular diseases, neurodegenerative disorders, and rheumatoid arthritis among others. Interestingly, these disorders were enriched significantly in the pathways related with the genes^17^.

Environment and diet can influence lifespan through several epigenetic mechanisms known to be altered during the normal aging process^18^. Epigenetic studies associated with frailty are still growing, and most of them have focused on DNA methylation patterns in blood samples. For instance, Bellizi *et al*.,^19^, found that the frailty status was associated with a significant decrease in the global DNA methylation levels. An apparent association between CpG island methylation and frailty was also found^20^. Other studies have also found a strong correlation between the epigenetic clock and frailty^21^. In this context, our findings are in the same direction, as there is evidence of epigenetic changes occurring at various levels, including reduced bulk levels of core histones, histone posttranslational modifications, replacement with histone variants and altered non-coding RNA molecules^22^. Moreover, our results showed that HIST1H3 cluster family genes and miR200 family could act both as hubs and bottlenecks. Dysregulation of HIST1H3 histone cluster family (which is particularly susceptible to acetylation)^23^, has been suggested as a driver mechanism of gliomas^24^. In addition, phosphorylation to H2A histone family member X (H2AX) could provide information regarding frailty severity^25^. On the other hand, posttranslational histone modifications do not affect DNA nucleotide sequence but can modify its availability to the transcriptional machinery. MiRNAs are a large family of small non-coding RNA’s that regulate gene expression post-transcriptionally via base pairing. Recently, they have been implicated in several regulatory networks relevant to aging, for instance, miR200 family which is highly expressed within epithelial cells and known to play crucial roles in cancer initiation and metastasis^24^, they have also been involved in atherosclerosis, Diabetes mellitus, kidney disease and other age-related diseases^26^.

Five well-defined clusters (Fig. 3) were recognized, regarding deficits associated with frailty and already mentioned genesets by Viña *et al*., and Hangelbroek *et al*.^14,15^. Cluster 1 corresponds to the homeostatic process of apoptosis signaling and immune system pathways and points to the possible link between frailty and “*inflammaging*” (i.e., the hypothesis that states that systemic increases of pro-inflammatory mediators are related with the frailty process, inducing metabolic dysfunction)^17^. A possible explanation of such is the induction of senescence-associated secretory phenotype (SASP), which contributes to damage of aging tissues and facilitates the progression of several age-related disorders.

Cluster 2 corresponds to genes related with Notch pathway, meaning that this transcription pathway could play a role in the development of frailty since CREBBP has an essential role in chromatin remodeling^27^; KAT2B regulate the balance between cell cycle arrest and apoptosis, and MTR activity is associated with hypomethylation and increased DNA damage^28^. Notch pathway defects have been reported in different human tissue, like muscles and brain diseases.

Regarding cluster 3, it includes genes involved in the central metabolic pathways of different tissues and organs, such as: striated muscle contraction, adrenaline and noradrenaline biosynthesis, cytoskeletal regulation, and nicotinic acetylcholine receptor signaling pathway. Alterations in these pathways can modify the physiology of the muscle tissue, consequently generating the development of sarcopenia. Interestingly, the signaling pathway ACVR1B/ACTN3/MSTN is related to the loss of skeletal muscle mass, and this may be correlated with cluster 1 since MSTN up-regulation involves TNF-α, suggesting a probable relationship between the inflammatory process and the development of muscular diseases^29^. Besides, MSTN inhibits the signaling pathway related to Insulin-like growth factor-1 (IGF-1) a potent muscle growth factor which progressively decreases with age, and has a crucial function in repairing and proliferation of skeletal muscle^29^. MSTN and TNF-α are also directly involved in the modification of the redox state of muscle tissue since their direct participation in the production of ROS have been reported through the activation of NADPH oxidase and the reduction of GST^30^. These have at least two consequences: oxidative damage of biomolecules that lead to apoptosis^29^ and a positive feedback mechanism that increases the expression of TNF-α and MSTN regulated by the activation of NF-κB a transcription factor sensitive to modifications of the cellular redox state^31^. Supplementary Figure 1S indicates the proposed mechanism for the proteins presented in gene sets and clusters based on reports of Sriram *et al*.^29,32^; Kovacheva *et al*., 2010^31^; and Zhou *et al*., 2016^33^.

Cluster 4 includes genes related to DNA metabolism and interestingly with Parkinson’s disease (PD). Frailty increases the vulnerability and impairs the homeostatic reserve, with the potential of further aggravation of functional deterioration in patients with PD. It also increases the risk of adverse consequences including falls, disability and death^16^. The molecular mechanisms underlying these interactions could be explained by their possible relationship with alterations in mitochondrial function, as reported by Gaare and collaborators in 2018.They demonstrated that the enrichment of rare inherited variation in the pathway controlling mitochondrial DNA replication and repair influenced the risk of PD and proposed that this polygenic enrichment contributes to the impairment of mitochondrial DNA homeostasis^34^, which could establish a relationship between mitochondrial dysfunction –characteristic of PD– and alterations in DNA metabolism.

Cluster 5 includes genes related to signaling and maintenance processes whose primary targets are the muscle and nervous system. It is well established that the lack of trophic inputs during adulthood leads to loss of physical function mediated by the loss of cell populations in different tissues. An example of this is the age-related reduced signaling of IGF-2, that leads to inefficient muscle regeneration. Ikemoto-Uezumi *et al*., 2015 reported that the protein expression of IGF-2 decreases the rate of muscle regeneration in the muscle of aged mice^34^. The ability to detect a decrease in the content of trophic factors or in the efficiency of the signaling pathways responsible for processing their stimulus could be promising for the timely detection of frailty. Overall, these findings support the statement of Neal S.F.^16^ and Viña J.^14^ that frailty is tightly associated with a failure in homeodynamics around apoptosis, energy metabolism (mitochondrial function), DNA integrity and neuromuscular changes.

Regarding the network pharmacology analysis; we identified compounds (Table 3) that could have a role in frailty. Some agents could be used because of their impact in inflammation (e.g., anti-rheumatic agents, in particular, small molecules coming from synthetic or natural sources), while others such as vitamin C or even valproate have beneficial effects on the regeneration of connective tissue –in particular in collagen sub-types. Moreover, endocrine pathways are also potential targets for pharmacological treatment, as shown by the estrogen-receptor blocker raloxifene; that is well-known for its impact on the treatment of osteoporosis and some types of cancer. Oxidative stress could also be halted by some of the candidate molecules found in the analysis; such as the epigallocatechin gallate (present in chocolate).

Further research must be assessed on the matter of repurposing drugs taking advantage of quantitative systems pharmacology (QSP), a novel tool useful to simulate drug efficacy (pharmacokinetics and pharmacodynamics) and toxicity for model-based design of treatment schedules to facilitate pharmaceutical development^35^. Drug repurposing has been shown to bring therapeutic alternatives for those conditions that still lack an effective treatment^36^. Our results, suggest that anti-rheumatic agents could have the potential to ameliorate the progression of frailty and even halt the burden it poses on older adults.

Finally, there are some interesting, albeit toxic compounds, that may interact with some of the most druggable targets GR and ACE, such as Bisphenol A (well-known endocrine disruptor chemical); which is present in several plastic bottles and metal cans linings that have shown to alter the epigenome. Also, arsenate, the most common form of inorganic arsenic and a widespread pollutant found in water, cigarettes, food, and air, that has been correlated with neurotoxic effects, high levels of DNA methylation and different types of cancer^34^. Altogether, these results suggest adverse environmental implications of frailty.

Our work highlights the potential that new methodologies could bring to the field of aging. Frailty in particular has shown to be a special case on the dynamics of a network of networks. Different levels, from the molecular to the clinical expression, seem to be related and understanding their mechanics, could improve knowledge both on causes and potential interventions. In conclusion, our results show several signaling pathways: apoptosis, inflammation, Notch, and chemokine signaling that play a significant role in the biology of frailty and may be considered as central hubs for this condition. Additionally, epigenetics may play a significant role in the development of frailty, highlighting the role of environmental factors such as pollution that could lead to the clinical expression of this problem. Finally, there may be some drugs that could be repurposed in order to be used in frailty, such as anti-rheumatic agents. However, further studies are needed to validate drug efficacy and toxicity before they could be implemented in clinical practice.

## Methods

### Genes related to frailty

In order to perform the interactome analysis, it was used the gene sets shown in Supplementary Table 1S, which have already been correlated with frailty by Viña *et al*., 2016^14^ and Hangelbroek *et al*., 2016^15^. Cytoscape software^37^ (v 3.7) was used to analyze, reconstruct, and perform structural analysis from all networks obtained in the analysis.

### Interactome analysis of frailty-related genes

Initially, we used a mirror version of GeneMANIA^38^ (a prediction server that finds functionally similar genes using a wealth of genomics and proteomics) on Cytoscape^39^ to obtain novel genes related by physical interactions, shared common signaling pathways and co-expression information of frailty-related genes (Fig. 1). Then we performed the enrichment analysis (see the section below) to analyze significant pathways and diseases from previously obtained genes (Table 1). For the second step, we used BisoGenet^40^ plug-in for Cytoscape to build a structural biological analysis based on PPI and miRNA-Protein interaction network analysis using as seeds (input values) only frailty-related genes^14,15^ (Fig. 2). The hubs (miRNA’s or proteins) of the network were detected using the cytoHubba plug-in developed for Cytoscape and node degree was chosen as a centrality index^41^. Node degree defined as the number of neighbors connected directly to a node. Nodes with the highest degrees considered as hubs and nodes with a higher number of betweenness and hub properties considered as bootlenecks^42^ (Table 2).

### Enrichment analysis

We performed an enrichment analysis of the pathways and diseases related with the genes previously obtained from GeneMANIA by using Comparative Toxicogenomics Database (CTD)^43^, Database for Annotation, Visualization, and Integrated Discovery (DAVID)^44^ and GSEAPY module from Python. The main biological process (BP, GO:0008150), as well as the main KEGG and Reactome pathways along with related diseases were chosen accordingly to the most significantly enriched terms, pathways and conditions (p value < 0.05, corrected by using the Benjamini-Hochberg procedure) and listed in Table 1 separated accordingly with pathways, gene ontology (GO)-terms and diseases.

### Genes-deficit analysis

Since the frailty index is a ratio of the number of deficits that an individual has accumulated divided by all deficits measured, we performed a search for the relationship between deficits associated with frailty and those already mentioned genes.

### Frailty definition and deficits

There is a reasonable consensus on what the conceptual definition of frailty is; however, an operational definition has been a matter of debate that persists to our days^2^. We chose the frailty index, since (Mitnitski *et al*., 2001)^45^ this tool allows us to look at different domains of this condition. In addition, it has shown that deficits interact as a network^46,47^ with particular characteristics that allow to approach this problem with a systems biology paradigm. These items are known as ‘deficits’, and the sum of many deficits (minimum 30 – Supplementary Table 3S) allows to give an individual a score of a frailty burden, in contrast to other tools that give cut-off points categorizing the subject as frail or not. The interest was to have a high number of features related to frailty in order to search for them in different databases; the frailty index suits this goal.

An exhaustive search of the different deficits that have been used to integrate a frailty index was done, departing from the first table provided by the creators of this tool, and enriched with other frailty indexes used by Mitniski *et al*.^48^ and Searle *et al*.^49^.

### Genes-deficit network construction

In order to relate already mentioned genes and deficits, it was developed a weighted socio-matrix or weighted adjacency matrix through the relation of original papers linking deficits with genes performed only on human samples or clinical studies on Pubmed over the last 15 years linking deficits with genes. The term socio-matrix is a square matrix where a number one indicates a tie between two nodes and a number zero indicates no tie. The convention is that rows indicate the starting node and columns the receiving node. In a weighted socio-matrix, like the one it was used, edge weights are included, and the number ones in the socio-matrix are substituted by the weights (publications) indicating the strength of a tie. Table 3S indicates the complete list of used MESH terms and names for the search. Supplementary Information Data 1 presents the constructed weighted socio-matrix. The number of papers linking deficit i; i = 1, …, 34, with a gene j; j = 1, …, 42, corresponds to the (i, j) entry of the socio-matrix, and by symmetry, the entry (j, i) has the same value. Associations between deficits on the one hand and between genes on the other were not considered; hence, we used zeros in the corresponding entries.

Networks were then built, and their nodes correspond to deficits or genes, and their edges are obtained from the weighted (publications) socio-matrix. Isolated vertices were eliminated. Centrality measures were calculated (i.e., degree, closeness and betweenness) to identify central nodes. The size of the nodes in the graphical representations depends on the value of the measure (Supplementary Figs 2S,3S). Since there are weights associated with each edge according to the socio-matrix, weighted centrality measures were calculated as well (Supplementary Fig. 3S).

To identify clustering formation k-cores (maximal subgraph, where each vertex is connected to at least k other vertices in the subgraph) were obtained (Fig. 4). For the same purpose, community detection algorithms were used (walktrap, edge betweenness, spin-glass, fast greedy, label propagation, leading eigenvector, and Louvain), and we chose the clusters obtained through the Louvain algorithm since it provided one of the most significant modularity values and better interpretability. For statistical analyses, software R was used (igraph, Intergraph, statnet, and tnet libraries). Additionally, clusters were enriched according to the metabolic pathways and the biological processes in which they participate (see Supplementary Table 4S).

### Network pharmacology

Once identified the most critical nodes on the deficit-gene network (Fig. 3), we performed network pharmacological analysis. In the first place, we analyzed the druggability score (evaluates the suitability of the binding site for small molecules under the Lipinski’s Rule of five); tractability score (similar to druggability score but under more relaxed conditions), and Ensemble score (the average of druggability score calculated under different models) from such nodes (Fig. 5). In the calculation of druggability, the average of the output from the specific predictions was calculated as the Ensemble Score ranging from undruggable (value of −1.0) to druggable (value of 1.00). Then a novel network to identify chemical targets (drugs or toxicants) that could interact somehow with the most druggable nodes was used by using the CTD (Fig. 5) with already known chemical compounds to identify those compounds that could be re-purposed and tested either alone or in combination in the treatment of frailty. The network was created by using Cytoscape (v 3.7.1), (Fig. 5 and Table 3) the complete list of all the compounds interacting with the primary targets is displayed in Supplementary Table 5S.

## Supplementary information

Supplementary Information

Supplementary Dataset
