# Supplementary material for: Systems biology and network pharmacology of frailty reveal novel epigenetic targets and mechanisms
Source: Sci Rep. 2019 Jul 22;9:10593. doi: 10.1038/s41598-019-47087-7 (PMC6646318; doi:10.1038/s41598-019-47087-7)
Supplement: Supplementary file 1 — Supplementary Information [file 41598_2019_47087_MOESM1_ESM.pdf]

Title

Systems biology and network pharmacology of frailty reveal novel epigenetic targets and mechanisms

Authors

[1]Gomez-Verjan JC .,[1]Ramirez-Aldana R., [1, 2]Pérez-Zepeda M.U., [1]Quiroz-Baez R., [1]Luna-Lopez A., [1]Gutierrez-Robledo L.M*.

Affiliations

[1]Instituto Nacional de Geriatría (INGER), Mexico City, Mexico

[2]Geriatric Medicine Research, Dalhousie University and Nova Scotia Health Authority, Halifax, NS, Canada

***Corresponding Author:** Dr.Gutiérrez-RobledoL.M.E-mail:gutierrezrobledoluismiguel@gmail.com, Anillo Periférico 2767, Magdalena Contreras, San Jerónimo Lídice, 10200 CDMX, Mexico.

Supplementary Tables pag. 3-53

Supplementary Appendix- Glossary of Terminology pag. -54-55

Supplementary Figures - pag. 56-58

KEYNOTES

There are significant pathways involved in frailty including apoptosis, proteolysis, muscle proliferation, and inflammation;

There are epigenetic factors as *HIST1H3* cluster and *miR200* family as hubs and bottlenecks in the interactome network

Connection between clinical deficits and genes, identified five clusters that give insights into the biology of frailty

Network pharmacology analysis of target nodes identified as potentially therapeutic compounds such as epigallocatechin gallate and antirheumatic agents.

Toxicants as bisphenols and arsenate may be involved in the development of this condition.

Supplementary Table 1S.Frailty-related genes list

**Genes**(Viña *et al.*, 2016) **Set-1**	**Genes**(Hangelbroek*et al.*, 2016) **Set-2**
IL-12,<br>ACVR1B,<br>MSTN,<br>IL-18,<br>SELP,<br>TNF,<br>IGF2,<br>ACTN3,<br>AMPD1,<br>LRP1,<br>ApoE,<br>CREBBP,<br>KAT2B,<br>CASP8,<br>MTR,<br>FN1,<br>GSTZ1,<br>TIAM1,<br>STAT1,<br>TCN2,<br>BTRC,<br>ACE,<br>CNTF,<br>GR	IGFN1,<br>UNC13C,<br>MYLK4,<br>C12orf75,<br>HCN1,<br>MYH8,<br>CFAP61,<br>NR4A3,<br>FAM83B,<br>DAAM2,<br>NNMT,<br>ZNF382,<br>TPPP3,<br>COL28A1,<br>METTL21EP,<br>HIST1H3E,<br>SERPINA5,<br>METTL21C

Supplementary Table 2S.miRNA´s list complete and network properties for each node							
**miRNA Name**	**Betweenness**	**BottleNeck**	**Closeness**	**Clustering Coefficient**	**Degree**	**Centricity**	**Radiality**
MIR200B	15667.97157	1	1348.83333	0.0287	147	0.24917	3.88384
MIR429	14552.65993	1	1335.75	0.02387	142	0.24917	3.85868
MIR130A	15307.68362	1	1326.5	0.0201	138	0.24917	3.84092
MIR200C	13919.53679	1	1344.5	0.02951	138	0.24917	3.88088
MIR130B	15093.55287	1	1322.16667	0.01818	134	0.24917	3.83426
MIR144	15454.71038	1	1327.08333	0.02695	132	0.24917	3.84684
MIR518E	13377.75748	5	1307.66667	0.02913	131	0.33222	3.80467
MIR200A	15947.58022	2	1307	0.01741	130	0.24917	3.80356
MIR518F	10335.03763	8	1293.08333	0.02628	129	0.24917	3.77322
MIR29C	14177.61256	1	1314.91667	0.02677	129	0.24917	3.82205
MIR519D	9822.52054	4	1311.16667	0.02562	127	0.33222	3.8154
MIR29A	12890.77935	1	1310.58333	0.02489	126	0.24917	3.81466
MIR132	10569.87668	1	1287.5	0.02	125	0.24917	3.76397
MIR518C	9491.75595	1	1302.25	0.03045	125	0.24917	3.7969
MIR95	15193.43793	1	1317.66667	0.02194	125	0.24917	3.83093
MIR27B	10503.202	1	1308.91667	0.0219	124	0.24917	3.81244
MIR27A	11664.86469	1	1303.08333	0.02218	121	0.24917	3.80171
MIR148A	9220.26493	1	1316.41667	0.03589	119	0.24917	3.83278
MIR518B	8656.83336	3	1302.75	0.02521	118	0.24917	3.80319
MIR454	10442.95085	1	1315.33333	0.02564	118	0.24917	3.83093
MIR922	11217.68473	1	1309.08333	0.02781	118	0.24917	3.81724
MIR19A	11769.92338	1	1267.58333	0.01709	117	0.24917	3.72549
MIR212	8470.45466	1	1276.5	0.02564	117	0.24917	3.74547
MIR181D	13198.02987	11	1289.08333	0.0148	115	0.24917	3.77507
MIR148B	9213.75156	1	1305.08333	0.02349	115	0.24917	3.81059
MIR34B	11265.22122	1	1280.83333	0.02487	115	0.24917	3.75657
MIR96	12843.80008	1	1269.16667	0.01522	114	0.33222	3.73178
MIR549A	10906.10038	2	1266.5	0.01628	113	0.24917	3.72623
MIR17	8753.94059	1	1315.25	0.02845	113	0.24917	3.83463
MIR198	9504.71201	1	1260.16667	0.01596	113	0.24917	3.71218
MIR221	9253.73436	1	1282.58333	0.02497	113	0.24917	3.76175
MIR367	10661.23121	1	1278	0.02481	113	0.24917	3.75176
MIR520G	7310.00327	1	1306.33333	0.03411	112	0.24917	3.8154
MIR20B	8347.84959	17	1314.75	0.03063	111	0.24917	3.835
MIR604	10159.18833	2	1304.66667	0.02735	111	0.33222	3.81281
MIR608	9821.40704	2	1259.41667	0.01736	111	0.24917	3.71181
MIR183	10142.53571	1	1269.33333	0.01753	111	0.24917	3.734
MIR214	9671.2573	1	1259	0.01343	111	0.33222	3.71144
MIR223	9874.31591	1	1275.91667	0.02569	110	0.24917	3.74917
MIR602	9453.42842	1	1249.91667	0.01468	110	0.24917	3.69183
MIR936	12051.93593	1	1292.08333	0.02335	110	0.24917	3.78543
MIR146A	8236.23422	1	1268	0.0248	109	0.24917	3.73252
MIR520H	7007.04274	1	1303.5	0.03092	109	0.24917	3.81133
MIR92B	9478.89216	1	1287.58333	0.02977	108	0.24917	3.77692

MIR99B	9418.91853	1	1281.66667	0.02769	108	0.24917	3.7636
MIR30D	8300.10068	1	1269.91667	0.02151	107	0.24917	3.73807
MIR335	7087.2514	1	1265.5	0.02786	107	0.24917	3.72845
MIR543	8617.05365	1	1264.83333	0.01763	107	0.24917	3.72697
MIR630	9146.67471	1	1275.16667	0.01816	107	0.24917	3.74991
MIR377	9056.86278	9	1254.83333	0.01833	106	0.24917	3.70552
MIR647	10225.4181	2	1328.58333	0.03845	106	0.24917	3.86941
MIR147B	7923.21713	1	1265.16667	0.02228	106	0.24917	3.72845
MIR648	10291.25105	1	1260.5	0.01509	106	0.24917	3.7181
MIR451A	8572.86949	9	1306.5	0.04286	105	0.24917	3.82094
MIR10A	8736.28995	1	1245.5	0.01447	105	0.24917	3.68554
MIR765	8700.01171	1	1256.08333	0.01667	105	0.24917	3.70885
MIR520E	7939.52299	7	1286.83333	0.02838	104	0.33222	3.7784
MIR493	9404.68983	7	1270.91667	0.02094	103	0.24917	3.74362
MIR663A	8380.26548	2	1246.58333	0.01333	103	0.24917	3.68924
MIR147A	9430.4036	1	1231.83333	0.01256	103	0.24917	3.65668
MIR22	10468.66892	1	1264.66667	0.02608	103	0.24917	3.72956
MIR222	6792.21219	1	1274.66667	0.0316	103	0.24917	3.75176
MIR25	7709.42857	1	1266.75	0.02303	103	0.24917	3.73437
MIR26B	7842.94664	1	1243.33333	0.01542	103	0.24917	3.68221
MIR99A	9082.9397	1	1261.75	0.02094	103	0.24917	3.72328
MIR376B	8735.09965	10	1281.41667	0.02271	102	0.24917	3.76767
MIR506	7989.03277	7	1252.08333	0.01805	102	0.24917	3.70256
MIR33B	9002.10737	2	1259.66667	0.01611	102	0.24917	3.71921
MIR564	8885.63516	2	1269.83333	0.01922	102	0.33222	3.74214
MIR100	7294.07926	1	1256.16667	0.02213	102	0.24917	3.71144
MIR10B	7822.98927	1	1248.58333	0.01922	102	0.24917	3.69479
MIR553	8182.673	1	1278.08333	0.02291	102	0.24917	3.76027
MIR573	10338.993	1	1290.83333	0.03145	102	0.33222	3.78876
MIR629	8506.33186	1	1247.33333	0.0167	102	0.24917	3.69183
MIR431	10523.34782	16	1236.83333	0.01267	101	0.24917	3.66926
MIR422A	8184.31793	4	1241.41667	0.01109	101	0.24917	3.67962
MIR301A	6156.65643	1	1290.5	0.02416	101	0.24917	3.78839
MIR589	9645.45907	1	1255.66667	0.01525	101	0.24917	3.71107
MIR638	10704.50317	1	1271.58333	0.02396	101	0.24917	3.74658
MIR33A	6875.39754	1	1259.5	0.01737	100	0.24917	3.72032
MIR657	8421.79699	1	1235.75	0.01111	100	0.24917	3.66778
MIR873	8064.81495	1	1273.5	0.02949	100	0.24917	3.75139
MIR877	10279.32945	1	1284	0.01778	100	0.33222	3.77507
MIR940	7714.16941	1	1231.91667	0.01273	100	0.24917	3.6589
MIR421	7680.57192	3	1277.33333	0.02762	99	0.24917	3.76064
MIR376C	7909.7754	1	1257	0.02268	99	0.24917	3.71551
MIR562	7059.95478	1	1250.58333	0.01113	99	0.24917	3.70145
MIR598	9516.43487	1	1257.66667	0.01938	99	0.24917	3.71699
MIR662	8674.41091	1	1302.41667	0.03979	99	0.24917	3.8165
MIR939	8759.84291	1	1253.08333	0.01464	99	0.24917	3.707
MIR137	8243.50813	2	1263.91667	0.02693	98	0.24917	3.73141

MIR892A	7433.08252	1	1234.5	0.01557	98	0.24917	3.6663
MIR921	9657.39955	1	1271.25	0.02125	98	0.24917	3.74806
MIR363	7006.57437	10	1261	0.02921	97	0.24917	3.72586
MIR519E	7922.04014	7	1261	0.01933	97	0.24917	3.72586
MIR552	9668.82407	2	1283.25	0.032	97	0.24917	3.77544
MIR649	6505.46323	2	1248.25	0.0174	97	0.24917	3.69738
MIR107	8060.99618	1	1256.08333	0.02448	97	0.24917	3.71514
MIR143	7220.28816	1	1281.75	0.02771	97	0.24917	3.77211
MIR155	7423.00214	1	1265.41667	0.02212	97	0.24917	3.73585
MIR328	8204.07178	1	1217.5	0.00773	97	0.24917	3.62931
MIR545	6207.44731	1	1280.91667	0.03157	97	0.24917	3.77026
MIRLET7D	5632.46307	1	1242.66667	0.01675	97	0.24917	3.68517
MIR888	8149.47202	1	1283.41667	0.03794	96	0.24917	3.77655
MIRLET7B	5789.59167	1	1259.83333	0.02215	96	0.24917	3.72401
MIR301B	5191.65987	1	1270.83333	0.02195	95	0.24917	3.74917
MIR650	7384.17574	1	1255.25	0.02396	95	0.24917	3.7144
MIR935	6413.9586	1	1274.33333	0.02979	95	0.33222	3.75731
MIR575	9855.6151	2	1275.08333	0.02768	94	0.24917	3.75953
MIR616	6914.07778	2	1245.83333	0.01876	94	0.24917	3.69442
MIR122	6842.54356	1	1228.25	0.0151	94	0.24917	3.65557
MIR541	8013.75563	1	1231	0.00961	94	0.24917	3.66149
MIR646	6922.71437	1	1248.66667	0.01624	94	0.24917	3.70071
MIR658	6436.28038	1	1243.33333	0.01533	94	0.24917	3.68887
MIR378A	6062.03825	12	1231.58333	0.01332	93	0.24917	3.66371
MIR496	9389.67736	6	1274.75	0.02992	93	0.24917	3.75953
MIR744	6613.90046	1	1246.16667	0.0187	93	0.24917	3.6959
MIRLET7E	5136.87398	1	1256.5	0.02197	93	0.24917	3.71884
MIR617	7870.5018	2	1231.83333	0.01839	92	0.24917	3.66482
MIR203A	7779.05438	1	1235.66667	0.01911	92	0.24917	3.67333
MIR802	9024.00687	1	1297.33333	0.02962	92	0.24917	3.81022
MIR920	7398.51832	1	1252.58333	0.01911	92	0.24917	3.71107
MIR937	7708.79704	1	1235.75	0.0215	92	0.24917	3.6737
MIR498	6007.54668	7	1267.25	0.02198	91	0.24917	3.74436
MIR606	6685.8112	2	1228.41667	0.01563	91	0.24917	3.65816
MIR618	8630.66335	2	1261.33333	0.02271	91	0.24917	3.73104
MIR215	7315.76785	1	1229.91667	0.01563	91	0.24917	3.66149
MIR217	8307.22961	1	1249.16667	0.01929	91	0.24917	3.70404
MIR297	9847.08807	1	1202.58333	0.00977	91	0.24917	3.60082
MIR634	8171.67472	1	1259.16667	0.02149	91	0.33222	3.7266
MIR487A	5815.13788	12	1205.33333	0.01099	90	0.24917	3.60748
MIR135B	6715.3085	1	1232.83333	0.01673	90	0.24917	3.66852
MIR185	7000.58201	1	1230.5	0.01199	90	0.24917	3.66334
MIR196B	6832.46126	1	1235.5	0.02147	90	0.24917	3.67444
MIR23A	6540.93198	1	1236.91667	0.02322	90	0.24917	3.67777
MIR570	5519.27401	1	1272.41667	0.03396	90	0.24917	3.75657
MIR611	8051.63777	1	1222.91667	0.01074	90	0.24917	3.64632
MIR659	9019.82893	1	1262.75	0.02022	90	0.24917	3.73511

MIR544A	6355.92648	2	1262	0.02477	89	0.24917	3.734
MIR205	5880.0731	1	1244.33333	0.02247	89	0.24917	3.69479
MIR554	6568.56066	1	1248.33333	0.02017	89	0.24917	3.70367
MIR890	6823.34521	1	1240.41667	0.02937	89	0.24917	3.68591
MIR591	8499.52592	2	1232.58333	0.01959	88	0.24917	3.66963
MIR182	7786.711	1	1264	0.02377	88	0.24917	3.73918
MIR216A	6454.11757	1	1214.5	0.01776	88	0.24917	3.62931
MIR571	7259.47247	1	1236.75	0.01855	88	0.24917	3.67888
MIR566	5660.5386	2	1244.58333	0.02513	87	0.24917	3.69664
MIR23B	6425.07823	1	1234.91667	0.02299	87	0.24917	3.67555
MIR370	7056.06842	1	1231.16667	0.0155	87	0.24917	3.66704
MIR411	6260.86504	1	1252	0.03261	87	0.33222	3.71366
MIR585	6748.09437	1	1221.5	0.01604	87	0.24917	3.64558
MIR586	7844.62149	2	1241.83333	0.02326	86	0.24917	3.69146
MIR134	6413.30193	1	1250.5	0.02709	86	0.33222	3.71107
MIR631	6482.24404	1	1225.83333	0.0156	86	0.24917	3.65594
MIR154	5052.99707	1	1223	0.02493	85	0.24917	3.65039
MIR210	6524.96649	1	1230.16667	0.02129	85	0.24917	3.6663
MIR760	6331.05275	1	1213.16667	0.01373	85	0.24917	3.62857
MIRLET7C	3657.94243	1	1235.16667	0.02213	85	0.24917	3.6774
MIR98	4482.00364	1	1231	0.01865	84	0.24917	3.66889
MIR492	7428.40835	8	1213.16667	0.01763	83	0.24917	3.63005
MIR579	6917.72015	2	1252.33333	0.01998	83	0.24917	3.71699
MIR192	6411.32566	1	1213.41667	0.01499	83	0.24917	3.63079
MIR551A	5362.86993	1	1197.83333	0.01381	83	0.24917	3.59601
MIR601	5365.32843	1	1204.83333	0.01322	83	0.24917	3.61155
MIR620	4912.27275	1	1212.91667	0.01763	83	0.24917	3.62931
MIR626	5813.04336	1	1174.75	0.01234	83	0.24917	3.54495
MIR933	6697.15771	1	1228.5	0.0241	83	0.24917	3.66408
MIR375	5382.02147	12	1237.33333	0.02349	82	0.24917	3.68443
MIR432	5490.63812	5	1205	0.01084	82	0.24917	3.61266
MIR510	6419.50145	5	1240.75	0.02529	82	0.24917	3.6922
MIR653	5679.64754	2	1239.66667	0.02409	82	0.24917	3.68961
MIR186	5864.23705	1	1216.75	0.01204	82	0.24917	3.63855
MIR340	5271.42847	1	1226.58333	0.01897	82	0.24917	3.66075
MIR644A	6229.32135	1	1207.75	0.01415	82	0.24917	3.61821
MIR551B	5402.63619	2	1234.33333	0.0287	81	0.24917	3.67851
MIR558	5247.54768	2	1199.58333	0.01327	81	0.24917	3.60119
MIR622	6216.46163	1	1246.83333	0.01481	81	0.24917	3.70626
MIR627	5471.36456	1	1255.16667	0.02469	81	0.24917	3.72475
MIR934	7168.64054	1	1249.08333	0.02037	81	0.24917	3.71144
MIR599	7507.60283	1	1248.58333	0.01646	80	0.24917	3.7107
MIR675	5987.07784	1	1190.75	0.00728	80	0.24917	3.58232
MIR595	5260.24784	1	1227.58333	0.0159	79	0.24917	3.66482
MIR944	5437.02377	1	1201.16667	0.01168	79	0.24917	3.60637
MIR410	4978.10498	6	1205.91667	0.01732	78	0.24917	3.61747
MIR539	4639.10432	1	1237.83333	0.02031	78	0.24917	3.6885

MIR588	4973.2071	1	1206.33333	0.01299	78	0.24917	3.61858
MIR612	4697.94896	1	1190.5	0.01572	77	0.24917	3.58306
MIR187	5514.98101	1	1242.41667	0.0207	76	0.24917	3.69997
MIR609	6816.55742	1	1209.41667	0.01053	76	0.24917	3.62709
MIR874	4119.31252	1	1189.66667	0.01684	76	0.24917	3.58306
MIR942	4695.01649	1	1226.5	0.02035	76	0.24917	3.66482
MIR197	5537.85285	2	1180	0.00865	75	0.24917	3.56234
MIR596	4379.30864	1	1196.5	0.01261	75	0.24917	3.59897
MIR636	5784.84695	1	1184.08333	0.00757	75	0.24917	3.57122
MIR190B	4752.24176	1	1195.66667	0.01407	74	0.24917	3.59786
MIR592	4986.53787	1	1239.08333	0.0137	74	0.24917	3.69442
MIR643	5170.89741	1	1221.33333	0.02332	74	0.24917	3.65483
MIR924	5699.99414	1	1220.91667	0.01888	74	0.24917	3.65372
MIR380	4443.61632	12	1264.41667	0.03044	73	0.24917	3.75139
MIR149	4846.22821	1	1189.5	0.01065	73	0.24917	3.58491
MIR580	4307.60492	1	1201.16667	0.01526	72	0.24917	3.61155
MIR635	5026.2696	1	1197.58333	0.0161	71	0.24917	3.60415
MIR639	3719.54887	1	1207.16667	0.01408	71	0.24917	3.62561
MIR504	4411.90247	4	1194.33333	0.01532	70	0.24917	3.59786
MIR581	5359.95395	2	1204.58333	0.01656	70	0.24917	3.62043
MIR559	4366.51822	1	1252.75	0.03023	70	0.24917	3.72771
MIR614	3593.49429	1	1195.33333	0.02387	69	0.24917	3.60082
MIR567	4330.84749	1	1211.83333	0.01888	68	0.24917	3.63818
MIR645	5978.16935	1	1254.08333	0.02657	66	0.24917	3.73363
MIR661	2679.69019	1	1158.25	0.01042	64	0.24917	3.52202
MIR665	3813.57117	1	1156.25	0.00694	64	0.24917	3.51758
MIR633	2973.18961	1	1168.91667	0.01469	60	0.24917	3.54865
MIR593	2678.79101	1	1154.41667	0.01512	58	0.24917	3.51721

Supplementary Table 3S. Frailty Deficits Names and MESH associated terms

Node Name	Deficit Name	Pubmed (MESH terms)
Everyday	Changes in everyday activities	Disability, loss of function
Headache	Headache	Cervicalgia, cephalea, headache, neck pain
Neck	Poor muscle tone in neck	Neck muscle paresis
Grooming	Problems getting dress, bathing, inconence, toilet, personal grooming	Disability, loss of function, dependence, activities of daily living
Rectal	Rectal problems	rectal diseases
Gastrointestinal	Gastrointestinal problems	Crohn`s disease, ulcerative colitis, Peptic ulcer disease, chronic diarrhea, celiac disease
Cooking_Shopping	Problems cooking, shopping, taking medication, going out alone	Disability, loss of function, dependence, instrumental activities of daily living
Sucking_problems	Sucking problems	Dysphagia
Mobility	Impaired mobility	Disability, loss of function, dependence, mobility impairment
Musculoskeletal problems	Musculoskeletal problems	Arthritis, osteoporosis
Ataxia	Poor limb coordination	Ataxia
Posture	Poor standing posture	Posture control
Gait	Irregular gait pattern	Gait
Falls	Falls	Falls

Mood	Mood problems, Depression	Depression
Tired	Tiredness at the time	Chronic fatigue syndrome, fibromyalgia
Sleep	Sleep changes	Sleep disorders
Restlessness	Restlessness	Anxiety, bipolar disorder
Cognitive	Memory changes, cognitive impairment, cognitive loss	dementia, cognitive impairment
Delirium	Clouding or delirium	delirium
Paranoid	Paranoid features	Psychotic symptoms
Neurologic	Impaired vibration, degenerative disease, snout reflex, palmomental reflex	Neurologic disorders
Parkinson	Parkinson´s disease	Parkinson`s disease, Tremor
Epileptic	Seizures, partial complex*<br>Seizures, generalized<br>Seizures, partial complex*<br>Seizures, generalized	Epileptic disorder (Epilepsy) , Tremor
Cerebrovascular	Cerebrovascular problems	Cerebrovascular diseases
Diabetes	Diabetes miellitus	Diabetes miellitus
Hypertension	Arterial hypertension	arterial hypertension
Vasculitis	Peripheral pulses	peripheral vascular disease, vasculitis

Cardiac	Cardiac problems, Myocardial infarction, Arrhytmia, Congestive heart failure	Heart failure,cardiomyopathies, angor pectoris
Lung	Lung problems, respiratory problems	Chronic obstructive pulmonary disease, pneumonia, asthma
Thyroid	Thyroid disease	Thyroid disease
Skin	Skin problems	dermatitis, herpes
Cancer	Malignant disease	Cancer
Breast	Breast problems	breast diseases

Supplementary Table 4S.Clusters of genes and deficits resulted from the network analysis using the associated sociomatrix

Cluster	Deficits	Gen	Pathway related to genes	Diseases related to gen
1	Headache<br>Gastrointestinal problems<br>Sucking problems<br>Poor limb coordination<br>Poor standing posture<br>Irregular gait pattern<br>Falls<br>Mood problems,<br>Depression<br>Tiredness all the time<br>Sleep changes<br>Clouding or delirium<br>Parkinson´s disease<br>Vasculitis<br>Skin problems	IL.12,<br>IL.18<br>TNF<br>ApoE<br>CASP8<br>ACE<br>GR	TNFR1-induced proapoptotic signaling, TNF signaling, apoptosis signaling pathway	Cerebrovascular Disorders, Miocardial ischemia, hemorragea, alzheimerdiesase, huntington disease
2	Memory changes, cognitive impairment, cognitive loss<br>Generalized, partial and complex Seizures<br>Arterial hypertension<br>Cardiac problems<br>Lung problems and respiratory problems<br>Thyroid disease	SELP<br>LRP1<br>CREBBP<br>KAT2B<br>MTR<br>FN1	Notch HLH transcription pathway, gonadotropin releasing hormone receptor pathway, p53 pathway, biosintesis de formyltetrahydroformate	Immune System disease, alzheimer disease-presenilin pathway, huntington disease
3	Changes in everyday activities<br>Problems getting dress, bathing, inconence, toilet, personal grooming<br>Problems cooking, shopping, taking medication, going out alone<br>Impaired mobility<br>Impaired vibration, degenerative disease, snout reflex, palmomental reflex<br>Diabetes miellitus<br>Cancer<br>Breast diseases	ACVR1B<br>MSTN<br>ACTN3<br>AMPD1<br>GSTZ1<br>TIAM1<br>BTRC<br>IGFN1<br>UNC13C<br>HCN1<br>MYH8<br>NR4A3<br>FAM83B<br>DAAM2<br>NNMT<br>ZNF382<br>TPPP3<br>COL28A1	Striated Muscle Contraction, adrenaline and noradrenaline biosynthesis, cytoskeletal regulation by RHO GTPase, nicoticnic acetylcholine receptor signaling pathway	Muscular diseases

		SERPINA5		
4	Parkinson´s disease	C12orf75<br>HIST1H3E	DNA replication, RNA polymerase I promoter opening	Parkinson´s disease
5	Rectal diseases<br>Musculoskeletal problems<br>Restlessness<br>Cerebrovascular problems	IGF2<br>STAT1<br>TCN2<br>CNTF<br>MYLK4<br>CFAP61<br>METTL21C	Interleukin-6 family signaling,<br>angioenesis,<br>EGF receptor singaling pathway,<br>inflamation mediated by chemokine	Nerve Degeneration, Anemia, Hemic and Lymphatic Diseases, Digestive System Neoplasms

Supplementary Table 5S. Target genes-compounds list complete

Compound	Target	Species	Type of Relation
1,1-bis(4-hydroxyphenyl)cyclohexane	NR3C1	Homo sapiens	1,1-bis(4-hydroxyphenyl)cyclohexane inhibits the reaction [Dexamethasone binds to NR3C1 protein]
1,2-bis(2-aminophenoxy)ethane N,N,N',N'-tetraacetic acid acetoxymethyl ester	NR3C1	Homo sapiens	1,2-bis(2-aminophenoxy)ethane N,N,N',N'-tetraacetic acid acetoxymethyl ester inhibits the reaction [Dexamethasone results in increased phosphorylation of NR3C1 protein]
1,4-dihydropyridine	NR3C1	Homo sapiens	1,4-dihydropyridine analog binds to and results in decreased activity of NR3C1 protein alternative form
15-deoxy-delta(12,14)-prostaglandin J2	NR3C1	Homo sapiens	15-deoxy-delta(12,14)-prostaglandin J2 affects the activity of NR3C1 protein
15-deoxy-delta(12,14)-prostaglandin J2	NR3C1	Homo sapiens	15-deoxy-delta(12,14)-prostaglandin J2 inhibits the reaction [Dexamethasone binds to NR3C1 protein]
15-deoxy-delta(12,14)-prostaglandin J2	NR3C1	Homo sapiens	15-deoxy-delta(12,14)-prostaglandin J2 inhibits the reaction [[Dexamethasone binds to NR3C1 protein] which results in decreased expression of CCL2 protein]
15-deoxyprostaglandin J2	NR3C1	Homo sapiens	15-deoxyprostaglandin J2 promotes the reaction [NR3C1 protein binds to PPARG protein]
15-deoxyprostaglandin J2	NR3C1	Homo sapiens	Fluticasone promotes the reaction [15-deoxyprostaglandin J2 promotes the reaction [NR3C1 protein binds to PPARG protein]]
15-deoxyprostaglandin J2	NR3C1	Homo sapiens	Salmeterol Xinafoate promotes the reaction [15-deoxyprostaglandin J2 promotes the reaction [NR3C1 protein binds to PPARG protein]]
17-alpha-hydroxy-progesterone caproate	NR3C1	Homo sapiens	17-alpha-hydroxy-progesterone caproate binds to and results in decreased activity of

			and results in increased localization of NR3C1 protein
17-alpha-hydroxy-progesterone caproate	NR3C1	Homo sapiens	17-alpha-hydroxy-progesterone caproate binds to and results in increased activity of NR3C1 protein
17-alpha-hydroxy-progesterone caproate	NR3C1	Homo sapiens	17-alpha-hydroxy-progesterone caproate inhibits the reaction [NR3C1 protein results in increased expression of FKBP5 mRNA]
17-alpha-hydroxy-progesterone caproate	NR3C1	Homo sapiens	17-alpha-hydroxy-progesterone caproate inhibits the reaction [NR3C1 protein results in increased expression of TSC22D3 mRNA]
2-(1H-indazol-4-yl)-6-(4-methanesulfonylpiperazin-1-ylmethyl)-4-morpholin-4-ylthieno(3,2-d)pyrimidine	NR3C1	Homo sapiens	[PIK3CA gene mutant form results in increased susceptibility to 2-(1H-indazol-4-yl)-6-(4-methanesulfonylpiperazin-1-ylmethyl)-4-morpholin-4-ylthieno(3,2-d)pyrimidine] which results in increased expression of NR3C1 mRNA
2,2',3,5',6-pentachlorobiphenyl	NR3C1	Homo sapiens	2,2',3,5',6-pentachlorobiphenyl inhibits the reaction [Budesonide results in increased activity of NR3C1 protein]
2,2',4,4',5-brominated diphenyl ether	NR3C1	Homo sapiens	2,2',4,4',5-brominated diphenyl ether inhibits the reaction [Hydrocortisone results in increased activity of NR3C1 protein]
2,2-bis(4-glycidyloxyphenyl)propane	NR3C1	Homo sapiens	2,2-bis(4-glycidyloxyphenyl)propane promotes the reaction [Dexamethasone binds to NR3C1 protein]
2,3',4,4',5-pentachlorobiphenyl	NR3C1	Homo sapiens	2,3',4,4',5-pentachlorobiphenyl inhibits the reaction [Budesonide results in increased activity of NR3C1 protein]
2,3',4,4',5-pentachlorobiphenyl	NR3C1	Homo sapiens	2,3',4,4',5-pentachlorobiphenyl results in increased activity of NR3C1 protein
2,4,2',4'-tetrachlorobiphenyl	NR3C1	Homo sapiens	2,4,2',4'-tetrachlorobiphenyl inhibits the reaction [Budesonide results in increased activity of NR3C1 protein]
2,4,4'-trichlorobiphenyl	NR3C1	Homo sapiens	2,4,4'-trichlorobiphenyl inhibits the reaction [Budesonide results in increased activity of NR3C1 protein]
2,4,5,2',4',5'-hexachlorobiphenyl	NR3C1	Homo sapiens	2,4,5,2',4',5'-hexachlorobiphenyl inhibits the reaction [Budesonide results in increased activity of NR3C1 protein]
2,4,5,2',4',5'-hexachlorobiphenyl	NR3C1	Homo sapiens	2,4,5,2',4',5'-hexachlorobiphenyl results in increased activity of NR3C1 protein
2,4,5,2',5'-pentachlorobiphenyl	NR3C1	Homo sapiens	2,4,5,2',5'-pentachlorobiphenyl inhibits the reaction [Budesonide results in increased activity of NR3C1 protein]
2,4,5,2',5'-pentachlorobiphenyl	NR3C1	Homo sapiens	2,4,5,2',5'-pentachlorobiphenyl metabolite inhibits the reaction [Dexamethasone results

			in increased activity of NR3C1 protein]
2,4,5,2',5'-pentachlorobiphenyl	NR3C1	Homo sapiens	2,4,5,2',5'-pentachlorobiphenyl results in increased activity of NR3C1 protein
2,4,6-trimethylbenzaldehyde	NR3C1	Homo sapiens	2,4,6-trimethylbenzaldehyde binds to and results in decreased activity of NR3C1 protein
2,5,2',5'-tetrachlorobiphenyl	NR3C1	Homo sapiens	2,5,2',5'-tetrachlorobiphenyl inhibits the reaction [Budesonide results in increased activity of NR3C1 protein]
2,5,2',5'-tetrachlorobiphenyl	NR3C1	Homo sapiens	2,5,2',5'-tetrachlorobiphenyl results in increased activity of NR3C1 protein
2,6-di-tert-butyl-4-hydroxy-4-methyl-2,5-cyclohexadien-1-one	NR3C1	Homo sapiens	2,6-di-tert-butyl-4-hydroxy-4-methyl-2,5-cyclohexadien-1-one binds to and results in decreased activity of NR3C1 protein
2-acetylphenothiazine	NR3C1	Homo sapiens	2-acetylphenothiazine inhibits the reaction [torcetrapib results in increased expression of NR3C1 mRNA]
2-aminophenol	NR3C1	Homo sapiens	NR3C1 gene SNP affects the susceptibility to 2-aminophenol
3,4,3',4'-tetrachloroazobenzene	NR3C1	Homo sapiens	3,4,3',4'-tetrachloroazobenzene binds to NR3C1 protein
3,4-dimethylbenzaldehyde	NR3C1	Homo sapiens	3,4-dimethylbenzaldehyde binds to and results in decreased activity of NR3C1 protein
3',5'-dichloro-2-hydroxy-2-methylbut-3-enanilide	NR3C1	Homo sapiens	3',5'-dichloro-2-hydroxy-2-methylbut-3-enanilide binds to and results in decreased activity of NR3C1 protein
3,5-di-tert-butyl-4-hydroxybenzaldehyde	NR3C1	Homo sapiens	3,5-di-tert-butyl-4-hydroxybenzaldehyde binds to and results in decreased activity of NR3C1 protein
3-phenoxybenzoic acid	NR3C1	Homo sapiens	3-phenoxybenzoic acid inhibits the reaction [Hydrocortisone results in increased activity of NR3C1 protein]
4,4'-bisphenol F	NR3C1	Homo sapiens	4,4'-bisphenol F inhibits the reaction [Dexamethasone binds to NR3C1 protein]
4,4'-bisphenol F	NR3C1	Homo sapiens	4,4'-bisphenol F inhibits the reaction [Dexamethasone results in increased activity of NR3C1 protein]
4-(5-benzo(1,3)dioxol-5-yl-4-pyridin-2-yl-1H-imidazol-2-yl)benzamide	NR3C1	Homo sapiens	[NOG protein co-treated with entinostat co-treated with (6-(4-(2-piperidin-1-ylethoxy)phenyl))-3-pyridin-4-ylpyrazolo(1,5-a)pyrimidine co-treated with 4-(5-benzo(1,3)dioxol-5-yl-4-pyridin-2-yl-1H-imidazol-2-yl)benzamide] results in increased expression of NR3C1 mRNA
4-(5-benzo(1,3)dioxol-5-yl-4-pyridin-2-yl-1H-imidazol-2-yl)benzamide	NR3C1	Homo sapiens	[NOG protein co-treated with mercuric bromide co-treated with (6-(4-(2-piperidin-1-ylethoxy)phenyl))-3-pyridin-4-ylpyrazolo(1,5-a)pyrimidine co-treated with 4-(5-

			benzo(1,3)dioxol-5-yl-4-pyridin-2-yl-1H-imidazol-2-yl)benzamide] results in increased expression of NR3C1 mRNA
4-(5-benzo(1,3)dioxol-5-yl-4-pyridin-2-yl-1H-imidazol-2-yl)benzamide	NR3C1	Homo sapiens	[NOG protein co-treated with Phenylmercuric Acetate co-treated with (6-(4-(2-piperidin-1-ylethoxy)phenyl))-3-pyridin-4-ylpyrazolo(1,5-a)pyrimidine co-treated with 4-(5-benzo(1,3)dioxol-5-yl-4-pyridin-2-yl-1H-imidazol-2-yl)benzamide] results in increased expression of NR3C1 mRNA
4-(5-benzo(1,3)dioxol-5-yl-4-pyridin-2-yl-1H-imidazol-2-yl)benzamide	NR3C1	Homo sapiens	[NOG protein co-treated with Valproic Acid co-treated with (6-(4-(2-piperidin-1-ylethoxy)phenyl))-3-pyridin-4-ylpyrazolo(1,5-a)pyrimidine co-treated with 4-(5-benzo(1,3)dioxol-5-yl-4-pyridin-2-yl-1H-imidazol-2-yl)benzamide] results in increased expression of NR3C1 mRNA
(6-(4-(2-piperidin-1-ylethoxy)phenyl))-3-pyridin-4-ylpyrazolo(1,5-a)pyrimidine	NR3C1	Homo sapiens	[NOG protein co-treated with entinostat co-treated with (6-(4-(2-piperidin-1-ylethoxy)phenyl))-3-pyridin-4-ylpyrazolo(1,5-a)pyrimidine co-treated with 4-(5-benzo(1,3)dioxol-5-yl-4-pyridin-2-yl-1H-imidazol-2-yl)benzamide] results in increased expression of NR3C1 mRNA
(6-(4-(2-piperidin-1-ylethoxy)phenyl))-3-pyridin-4-ylpyrazolo(1,5-a)pyrimidine	NR3C1	Homo sapiens	[NOG protein co-treated with mercuric bromide co-treated with (6-(4-(2-piperidin-1-ylethoxy)phenyl))-3-pyridin-4-ylpyrazolo(1,5-a)pyrimidine co-treated with 4-(5-benzo(1,3)dioxol-5-yl-4-pyridin-2-yl-1H-imidazol-2-yl)benzamide] results in increased expression of NR3C1 mRNA
(6-(4-(2-piperidin-1-ylethoxy)phenyl))-3-pyridin-4-ylpyrazolo(1,5-a)pyrimidine	NR3C1	Homo sapiens	[NOG protein co-treated with Phenylmercuric Acetate co-treated with (6-(4-(2-piperidin-1-ylethoxy)phenyl))-3-pyridin-4-ylpyrazolo(1,5-a)pyrimidine co-treated with 4-(5-benzo(1,3)dioxol-5-yl-4-pyridin-2-yl-1H-imidazol-2-yl)benzamide] results in increased expression of NR3C1 mRNA
(6-(4-(2-piperidin-1-ylethoxy)phenyl))-3-pyridin-4-ylpyrazolo(1,5-a)pyrimidine	NR3C1	Homo sapiens	[NOG protein co-treated with Valproic Acid co-treated with (6-(4-(2-piperidin-1-ylethoxy)phenyl))-3-pyridin-4-ylpyrazolo(1,5-a)pyrimidine co-treated with 4-(5-benzo(1,3)dioxol-5-yl-4-pyridin-2-yl-1H-imidazol-2-yl)benzamide] results in increased expression of NR3C1 mRNA
9,10-dihydro-9,10-dihydroxybenzo(a)pyrene	NR3C1	Homo sapiens	9,10-dihydro-9,10-dihydroxybenzo(a)pyrene results in increased expression of NR3C1 mRNA

Acetaminophen	NR3C 1	Homo sapiens	Acetaminophen results in decreased expression of NR3C1 mRNA
Acetaminophen	NR3C 1	Homo sapiens	Acetaminophen results in increased expression of NR3C1 mRNA
Acetylcysteine	NR3C 1	Homo sapiens	Acetylcysteine inhibits the reaction [anacetrapib results in increased expression of NR3C1 mRNA]
Acetylcysteine	NR3C 1	Homo sapiens	Acetylcysteine inhibits the reaction [dalcetrapib results in increased expression of NR3C1 mRNA]
Acetylcysteine	NR3C 1	Homo sapiens	Acetylcysteine inhibits the reaction [torcetrapib results in increased expression of NR3C1 mRNA]
Acetylcysteine	NR3C 1	Homo sapiens	Acetylcysteine results in decreased expression of NR3C1 mRNA
aclacinomycins	NR3C 1	Homo sapiens	aclacinomycins results in decreased activity of NR3C1 protein
Adrenocorticotropic Hormone	NR3C 1	Homo sapiens	NR3C1 gene polymorphism affects the abundance of Adrenocorticotropic Hormone
Aflatoxin B1	NR3C 1	Homo sapiens	Aflatoxin B1 results in decreased methylation of NR3C1 gene
Albuterol	NR3C 1	Homo sapiens	[Beclomethasone co-treated with Albuterol] affects the localization of NR3C1 protein
Aldosterone	NR3C 1	Homo sapiens	Aldosterone binds to and results in increased activity of NR3C1 protein
Aldosterone	NR3C 1	Homo sapiens	Aldosterone binds to NR3C1 protein mutant form
Aldosterone	NR3C 1	Homo sapiens	Aldosterone results in increased activity of NR3C1 protein
anacetrapib	NR3C 1	Homo sapiens	Acetylcysteine inhibits the reaction [anacetrapib results in increased expression of NR3C1 mRNA]
anacetrapib	NR3C 1	Homo sapiens	anacetrapib results in increased expression of NR3C1 mRNA
anacetrapib	NR3C 1	Homo sapiens	GKT137831 inhibits the reaction [anacetrapib results in increased expression of NR3C1 mRNA]
Antirheumatic Agents	NR3C 1	Homo sapiens	Antirheumatic Agents results in increased expression of NR3C1 mRNA
arsenic trioxide	NR3C 1	Homo sapiens	arsenic trioxide affects the reaction [NR3C1 protein modified form binds to DAXX protein]
arsenic trioxide	NR3C 1	Homo sapiens	arsenic trioxide inhibits the reaction [[DAXX protein binds to NR3C1 protein] which binds to TSC22D3 promoter]
arsenite	NR3C 1	Homo sapiens	arsenite inhibits the reaction [Dexamethasone binds to NR3C1 protein]
arsenite	NR3C	Homo	Dithiothreitol inhibits the reaction [arsenite

	1	sapiens	inhibits the reaction [Dexamethasone binds to NR3C1 protein]]
Ascorbic Acid	NR3C1	Homo sapiens	[Tretinoin co-treated with Ascorbic Acid] results in increased expression of NR3C1 mRNA
Atrazine	NR3C1	Homo sapiens	Atrazine inhibits the reaction [Hydrocortisone results in increased activity of NR3C1 protein]
aurin	NR3C1	Homo sapiens	aurin inhibits the reaction [NR3C1 protein results in increased expression of FKBP5 mRNA]
aurin	NR3C1	Homo sapiens	aurin results in decreased activity of NR3C1 protein
avobenzone	NR3C1	Homo sapiens	avobenzone binds to NR3C1 protein
Aziridines	NR3C1	Homo sapiens	NR3C1 protein promotes the reaction [Aziridines analog inhibits the reaction [TNF protein results in increased expression of IL6 mRNA]]
Beclomethasone	NR3C1	Homo sapiens	Beclomethasone affects the localization of and results in increased activity of NR3C1 protein
Beclomethasone	NR3C1	Homo sapiens	Beclomethasone affects the localization of NR3C1 protein
Beclomethasone	NR3C1	Homo sapiens	Beclomethasone binds to and results in increased activity of NR3C1 protein
Beclomethasone	NR3C1	Homo sapiens	Beclomethasone binds to NR3C1 protein
Beclomethasone	NR3C1	Homo sapiens	[Beclomethasone co-treated with Albuterol] affects the localization of NR3C1 protein
Beclomethasone	NR3C1	Homo sapiens	[Beclomethasone co-treated with Formoterol Fumarate] affects the localization of NR3C1 protein
Beclomethasone	NR3C1	Homo sapiens	Beclomethasone results in increased activity of NR3C1 protein
Beclomethasone	NR3C1	Homo sapiens	Beclomethasone results in increased expression of NR3C1 mRNA
beclomethasone 17-monopropionate	NR3C1	Homo sapiens	beclomethasone 17-monopropionate binds to NR3C1 protein
beclomethasone 17-monopropionate	NR3C1	Homo sapiens	NR3C1 mRNA promotes the reaction [beclomethasone 17-monopropionate results in increased expression of CYP3A5 mRNA]
Benzene	NR3C1	Homo sapiens	Benzene analog binds to and results in decreased activity of NR3C1 protein
Benzo(a)pyrene	NR3C1	Homo sapiens	Benzo(a)pyrene affects the activity of NR3C1 protein
Benzo(a)pyrene	NR3C1	Homo sapiens	Benzo(a)pyrene affects the reaction [Dexamethasone affects the expression of

			NR3C1 mRNA]
Benzo(a)pyrene	NR3C1	Homo sapiens	Benzo(a)pyrene results in increased expression of NR3C1 mRNA
7,8-Dihydro-7,8-dihydroxybenzo(a)pyrene 9,10-oxide	NR3C1	Homo sapiens	7,8-Dihydro-7,8-dihydroxybenzo(a)pyrene 9,10-oxide results in decreased expression of NR3C1 mRNA
7,8-Dihydro-7,8-dihydroxybenzo(a)pyrene 9,10-oxide	NR3C1	Homo sapiens	7,8-Dihydro-7,8-dihydroxybenzo(a)pyrene 9,10-oxide results in increased expression of NR3C1 mRNA
benzyloxycarbonylleucyl-leucyl-leucine aldehyde	NR3C1	Homo sapiens	benzyloxycarbonylleucyl-leucyl-leucine aldehyde inhibits the reaction [Dexamethasone results in increased degradation of NR3C1 protein]
Estradiol	NR3C1	Homo sapiens	Dexamethasone promotes the reaction [Estradiol results in decreased expression of NR3C1 mRNA]
Estradiol	NR3C1	Homo sapiens	[Estradiol co-treated with Dexamethasone] inhibits the reaction [NR3C1 protein binds to TSC22D3 promoter]
Estradiol	NR3C1	Homo sapiens	Estradiol results in decreased expression of NR3C1 mRNA
Estradiol	NR3C1	Homo sapiens	NR3C1 protein inhibits the reaction [[Progesterone co-treated with 8-Bromo Cyclic Adenosine Monophosphate co-treated with Estradiol] results in decreased expression of GRIA1 mRNA]
Estradiol	NR3C1	Homo sapiens	NR3C1 protein inhibits the reaction [[Progesterone co-treated with 8-Bromo Cyclic Adenosine Monophosphate co-treated with Estradiol] results in increased expression of ZNF486 mRNA]
Estradiol	NR3C1	Homo sapiens	NR3C1 protein promotes the reaction [[Progesterone co-treated with 8-Bromo Cyclic Adenosine Monophosphate co-treated with Estradiol] results in increased expression of WNT4 mRNA]
Estradiol	NR3C1	Homo sapiens	[Progesterone co-treated with 8-Bromo Cyclic Adenosine Monophosphate co-treated with Estradiol] results in decreased expression of NR3C1 protein
Betamethasone	NR3C1	Homo sapiens	Betamethasone binds to NR3C1 protein
Betamethasone	NR3C1	Homo sapiens	Betamethasone results in increased activity of NR3C1 protein
Betamethasone	NR3C1	Homo sapiens	NR3C1 protein alternative form inhibits the reaction [Betamethasone results in increased activity of NR3C1 protein alternative form]

bifenthrin	NR3C 1	Homo sapiens	bifenthrin inhibits the reaction [Hydrocortisone results in increased activity of NR3C1 protein]
bis(4-hydroxyphenyl)sulfone	NR3C 1	Homo sapiens	bis(4-hydroxyphenyl)sulfone inhibits the reaction [Dexamethasone binds to NR3C1 protein]
bisphenol A	NR3C 1	Homo sapiens	bisphenol A analog inhibits the reaction [Dexamethasone binds to NR3C1 protein]
bisphenol A	NR3C 1	Homo sapiens	bisphenol A inhibits the reaction [Dexamethasone binds to NR3C1 protein]
bisphenol A	NR3C 1	Homo sapiens	bisphenol A inhibits the reaction [Dexamethasone results in increased activity of NR3C1 protein]
bisphenol A	NR3C 1	Homo sapiens	bisphenol A results in decreased activity of NR3C1 protein
bisphenol A	NR3C 1	Homo sapiens	NR3C1 protein promotes the reaction [bisphenol A results in decreased expression of SCNN1G protein]
bisphenol B	NR3C 1	Homo sapiens	bisphenol B inhibits the reaction [Dexamethasone binds to NR3C1 protein]
8-Bromo Cyclic Adenosine Monophosphate	NR3C 1	Homo sapiens	[Medroxyprogesterone Acetate co-treated with 8-Bromo Cyclic Adenosine Monophosphate] results in decreased expression of NR3C1 mRNA
8-Bromo Cyclic Adenosine Monophosphate	NR3C 1	Homo sapiens	[Medroxyprogesterone Acetate co-treated with 8-Bromo Cyclic Adenosine Monophosphate] results in decreased expression of NR3C1 protein
8-Bromo Cyclic Adenosine Monophosphate	NR3C 1	Homo sapiens	NR3C1 protein inhibits the reaction [[Progesterone co-treated with 8-Bromo Cyclic Adenosine Monophosphate co-treated with Estradiol] results in decreased expression of GRIA1 mRNA]
8-Bromo Cyclic Adenosine Monophosphate	NR3C 1	Homo sapiens	NR3C1 protein inhibits the reaction [[Progesterone co-treated with 8-Bromo Cyclic Adenosine Monophosphate co-treated with Estradiol] results in increased expression of ZNF486 mRNA]
8-Bromo Cyclic Adenosine Monophosphate	NR3C 1	Homo sapiens	NR3C1 protein promotes the reaction [[Progesterone co-treated with 8-Bromo Cyclic Adenosine Monophosphate co-treated with Estradiol] results in increased expression of WNT4 mRNA]
8-Bromo Cyclic Adenosine Monophosphate	NR3C 1	Homo sapiens	[Progesterone co-treated with 8-Bromo Cyclic Adenosine Monophosphate co-treated with Estradiol] results in decreased expression of NR3C1 protein

8-Bromo Cyclic Adenosine Monophosphate	NR3C 1	Homo sapiens	[Progesterone co-treated with 8-Bromo Cyclic Adenosine Monophosphate] results in decreased expression of NR3C1 mRNA
Budesonide	NR3C 1	Homo sapiens	2,2',3,5',6-pentachlorobiphenyl inhibits the reaction [Budesonide results in increased activity of NR3C1 protein]
Budesonide	NR3C 1	Homo sapiens	2,3',4,4',5-pentachlorobiphenyl inhibits the reaction [Budesonide results in increased activity of NR3C1 protein]
Budesonide	NR3C 1	Homo sapiens	2,4,2',4'-tetrachlorobiphenyl inhibits the reaction [Budesonide results in increased activity of NR3C1 protein]
Budesonide	NR3C 1	Homo sapiens	2,4,4'-trichlorobiphenyl inhibits the reaction [Budesonide results in increased activity of NR3C1 protein]
Budesonide	NR3C 1	Homo sapiens	2,4,5,2',4',5'-hexachlorobiphenyl inhibits the reaction [Budesonide results in increased activity of NR3C1 protein]
Budesonide	NR3C 1	Homo sapiens	2,4,5,2',5'-pentachlorobiphenyl inhibits the reaction [Budesonide results in increased activity of NR3C1 protein]
Budesonide	NR3C 1	Homo sapiens	2,5,2',5'-tetrachlorobiphenyl inhibits the reaction [Budesonide results in increased activity of NR3C1 protein]
Budesonide	NR3C 1	Homo sapiens	Budesonide affects the reaction [Terbutaline results in increased expression of NR3C1 mRNA]
Budesonide	NR3C 1	Homo sapiens	Budesonide binds to NR3C1 protein
Budesonide	NR3C 1	Homo sapiens	Budesonide results in decreased expression of NR3C1 mRNA
Budesonide	NR3C 1	Homo sapiens	Budesonide results in increased activity of NR3C1 protein
butachlor	NR3C 1	Homo sapiens	butachlor results in increased activity of NR3C1 protein
tert-Butylhydroperoxide	NR3C 1	Homo sapiens	tert-Butylhydroperoxide affects the expression of NR3C1 mRNA
butylparaben	NR3C 1	Homo sapiens	[butylparaben co-treated with propylparaben] results in increased activity of NR3C1 protein
butylparaben	NR3C 1	Homo sapiens	butylparaben results in increased activity of NR3C1 protein
butylparaben	NR3C 1	Homo sapiens	[Diethylhexyl Phthalate co-treated with butylparaben co-treated with propylparaben] results in increased activity of NR3C1 protein
butylparaben	NR3C 1	Homo sapiens	[Diethylhexyl Phthalate co-treated with butylparaben co-treated with tetramethrin co-treated with propylparaben] results in

			increased activity of NR3C1 protein
butylparaben	NR3C 1	Homo sapiens	[Diethylhexyl Phthalate co-treated with butylparaben co-treated with tetramethrin] results in increased activity of NR3C1 protein
butylparaben	NR3C 1	Homo sapiens	[Diethylhexyl Phthalate co-treated with butylparaben] results in increased activity of NR3C1 protein
butyraldehyde	NR3C 1	Homo sapiens	butyraldehyde results in decreased expression of NR3C1 mRNA
Butyrates	NR3C 1	Homo sapiens	Butyrates results in decreased expression of NR3C1 mRNA alternative form
Butyrates	NR3C 1	Homo sapiens	Butyrates results in decreased expression of NR3C1 protein alternative form
Butyrates	NR3C 1	Homo sapiens	Butyrates results in increased expression of NR3C1 mRNA alternative form
Butyrates	NR3C 1	Homo sapiens	Butyrates results in increased expression of NR3C1 protein alternative form
Cadmium	NR3C 1	Homo sapiens	Cadmium inhibits the reaction [Dexamethasone binds to NR3C1 protein]
Cadmium Chloride	NR3C 1	Homo sapiens	Cadmium Chloride results in increased expression of NR3C1 mRNA
Carbamazepine	NR3C 1	Homo sapiens	Carbamazepine affects the expression of NR3C1 mRNA
Carbamazepine	NR3C 1	Homo sapiens	Carbamazepine results in increased expression of NR3C1 mRNA
Carbamazepine	NR3C 1	Homo sapiens	NR3C1 protein promotes the reaction [Carbamazepine results in increased expression of CYP3A4 mRNA]
chromium hexavalent ion	NR3C 1	Homo sapiens	chromium hexavalent ion results in decreased expression of NR3C1 protein
ciclopirox	NR3C 1	Homo sapiens	ciclopirox results in decreased activity of NR3C1 protein
ciclopirox	NR3C 1	Homo sapiens	ciclopirox results in decreased expression of NR3C1 protein
cidofovir	NR3C 1	Homo sapiens	cidofovir results in decreased expression of NR3C1 mRNA
cinerubine A	NR3C 1	Homo sapiens	cinerubine A results in decreased activity of NR3C1 protein
cinerubine B	NR3C 1	Homo sapiens	cinerubine B results in decreased activity of NR3C1 protein
Citalopram	NR3C 1	Homo sapiens	Citalopram inhibits the reaction [Dexamethasone results in increased expression of NR3C1 mRNA]
Clodronic Acid	NR3C 1	Homo sapiens	Clodronic Acid results in decreased expression of NR3C1 mRNA
Clotrimazole	NR3C 1	Homo sapiens	Dexamethasone promotes the reaction [NR3C1 protein affects the reaction [NR1I2

			protein promotes the reaction [Clotrimazole results in increased expression of CYP2B6 mRNA]]]
Cocaine	NR3C 1	Homo sapiens	Cocaine results in decreased expression of NR3C1 mRNA
Colchicine	NR3C 1	Homo sapiens	Colchicine results in decreased localization of and results in decreased activity of NR3C1 protein
Colchicine	NR3C 1	Homo sapiens	Colchicine results in increased activity of NR3C1 protein
Colchicine	NR3C 1	Homo sapiens	Colchicine results in increased degradation of NR3C1 protein
Colforsin	NR3C 1	Homo sapiens	Colforsin results in increased activity of NR3C1 protein
Copper Sulfate	NR3C 1	Homo sapiens	Copper Sulfate results in increased expression of NR3C1 mRNA
Corticosterone	NR3C 1	Homo sapiens	Corticosterone binds to and results in increased activity of NR3C1 protein
Corticosterone	NR3C 1	Homo sapiens	Corticosterone binds to NR3C1 protein
Corticosterone	NR3C 1	Homo sapiens	Corticosterone results in increased activity of NR3C1 protein
Corticosterone	NR3C 1	Homo sapiens	Corticosterone results in increased expression of NR3C1 mRNA
Cortisone	NR3C 1	Homo sapiens	Cortisone binds to NR3C1 protein
Cortisone	NR3C 1	Homo sapiens	Cortisone results in increased activity of NR3C1 protein
Cortisone	NR3C 1	Homo sapiens	[Mifepristone binds to and results in decreased activity of NR3C1 protein] inhibits the reaction [[HSD11B1 protein results in increased activity of Cortisone] inhibits the reaction [sulforafan results in increased activity of NFE2L2 protein]]
cortivazol	NR3C 1	Homo sapiens	cortivazol binds to and results in increased activity of NR3C1 protein
Coumestrol	NR3C 1	Homo sapiens	[Coumestrol co-treated with resveratrol] results in decreased expression of NR3C1 mRNA
Coumestrol	NR3C 1	Homo sapiens	Coumestrol results in decreased expression of NR3C1 mRNA
croneton	NR3C 1	Homo sapiens	croneton inhibits the reaction [Hydrocortisone results in increased activity of NR3C1 protein]
Curcumin	NR3C 1	Homo sapiens	Curcumin inhibits the reaction [Dexamethasone results in increased phosphorylation of NR3C1 protein]
Cyclosporine	NR3C	Homo	Cyclosporine results in increased expression

	1	sapiens	of NR3C1 mRNA
cyhalothrin	NR3C 1	Homo sapiens	cyhalothrin inhibits the reaction [Hydrocortisone results in increased activity of NR3C1 protein]
cypermethrin	NR3C 1	Homo sapiens	cypermethrin inhibits the reaction [Hydrocortisone results in increased activity of NR3C1 protein]
dalcetrapib	NR3C 1	Homo sapiens	Acetylcysteine inhibits the reaction [dalcetrapib results in increased expression of NR3C1 mRNA]
dalcetrapib	NR3C 1	Homo sapiens	dalcetrapib results in increased expression of NR3C1 mRNA
Daunorubicin	NR3C 1	Homo sapiens	Daunorubicin results in decreased activity of NR3C1 protein
DDT	NR3C 1	Homo sapiens	DDT inhibits the reaction [Hydrocortisone results in increased activity of NR3C1 protein]
deacylcortivazol	NR3C 1	Homo sapiens	deacylcortivazol promotes the reaction [MAPK14 protein results in increased phosphorylation of NR3C1 protein]
deacylcortivazol	NR3C 1	Homo sapiens	deacylcortivazol results in increased phosphorylation of NR3C1 protein
deacylcortivazol	NR3C 1	Homo sapiens	SB 203580 inhibits the reaction [deacylcortivazol results in increased phosphorylation of NR3C1 protein]
decitabine	NR3C 1	Homo sapiens	decitabine results in decreased expression of NR3C1 mRNA alternative form
decitabine	NR3C 1	Homo sapiens	decitabine results in decreased expression of NR3C1 protein alternative form
decitabine	NR3C 1	Homo sapiens	decitabine results in increased expression of NR3C1 mRNA alternative form
decitabine	NR3C 1	Homo sapiens	decitabine results in increased expression of NR3C1 protein alternative form
deflazacort	NR3C 1	Homo sapiens	deflazacort results in increased activity of NR3C1 protein
Dehydroepiandrosterone	NR3C 1	Homo sapiens	Dehydroepiandrosterone results in increased expression of NR3C1 protein
Dehydroepiandrosterone	NR3C 1	Homo sapiens	Flutamide inhibits the reaction [Dehydroepiandrosterone results in increased expression of NR3C1 protein]
Cortodoxone	NR3C 1	Homo sapiens	Cortodoxone binds to NR3C1 protein
Desipramine	NR3C 1	Homo sapiens	Desipramine inhibits the reaction [IFNA1 protein results in decreased expression of NR3C1 mRNA]
Desipramine	NR3C 1	Homo sapiens	Desipramine inhibits the reaction [IFNA1 protein results in decreased expression of NR3C1 protein]

desisobutyrylciclesonide	NR3C1	Homo sapiens	desisobutyrylciclesonide binds to NR3C1 protein
Dexamethasone	NR3C1	Homo sapiens	1,1-bis(4-hydroxyphenyl)cyclohexane inhibits the reaction [Dexamethasone binds to NR3C1 protein]
Dexamethasone	NR3C1	Homo sapiens	1,2-bis(2-aminophenoxy)ethane N,N,N',N'-tetraacetic acid acetoxymethyl ester inhibits the reaction [Dexamethasone results in increased phosphorylation of NR3C1 protein]
Dexamethasone	NR3C1	Homo sapiens	15-deoxy-delta(12,14)-prostaglandin J2 inhibits the reaction [Dexamethasone binds to NR3C1 protein]
Dexamethasone	NR3C1	Homo sapiens	15-deoxy-delta(12,14)-prostaglandin J2 inhibits the reaction [[Dexamethasone binds to NR3C1 protein] which results in decreased expression of CCL2 protein]
Dexamethasone	NR3C1	Homo sapiens	2,2-bis(4-glycidyloxyphenyl)propane promotes the reaction [Dexamethasone binds to NR3C1 protein]
Dexamethasone	NR3C1	Homo sapiens	2,4,5,2',5'-pentachlorobiphenyl metabolite inhibits the reaction [Dexamethasone results in increased activity of NR3C1 protein]
Dexamethasone	NR3C1	Homo sapiens	4,4'-bisphenol F inhibits the reaction [Dexamethasone binds to NR3C1 protein]
Dexamethasone	NR3C1	Homo sapiens	4,4'-bisphenol F inhibits the reaction [Dexamethasone results in increased activity of NR3C1 protein]
Dexamethasone	NR3C1	Homo sapiens	arsenite inhibits the reaction [Dexamethasone binds to NR3C1 protein]
Dexamethasone	NR3C1	Homo sapiens	Benzo(a)pyrene affects the reaction [Dexamethasone affects the expression of NR3C1 mRNA]
Dexamethasone	NR3C1	Homo sapiens	benzyloxycarbonylleucyl-leucyl-leucine aldehyde inhibits the reaction [Dexamethasone results in increased degradation of NR3C1 protein]
Dexamethasone	NR3C1	Homo sapiens	bis(4-hydroxyphenyl)sulfone inhibits the reaction [Dexamethasone binds to NR3C1 protein]
Dexamethasone	NR3C1	Homo sapiens	bisphenol A analog inhibits the reaction [Dexamethasone binds to NR3C1 protein]
Dexamethasone	NR3C1	Homo sapiens	bisphenol A inhibits the reaction [Dexamethasone binds to NR3C1 protein]
Dexamethasone	NR3C1	Homo sapiens	bisphenol A inhibits the reaction [Dexamethasone results in increased activity of NR3C1 protein]
Dexamethasone	NR3C	Homo	bisphenol B inhibits the reaction

	1	sapiens	[Dexamethasone binds to NR3C1 protein]
Dexamethasone	NR3C 1	Homo sapiens	Cadmium inhibits the reaction [Dexamethasone binds to NR3C1 protein]
Dexamethasone	NR3C 1	Homo sapiens	Citalopram inhibits the reaction [Dexamethasone results in increased expression of NR3C1 mRNA]
Dexamethasone	NR3C 1	Homo sapiens	Curcumin inhibits the reaction [Dexamethasone results in increased phosphorylation of NR3C1 protein]
Dexamethasone	NR3C 1	Homo sapiens	Dexamethasone affects the expression of NR3C1 mRNA
Dexamethasone	NR3C 1	Homo sapiens	Dexamethasone binds to and affects the localization of NR3C1 protein
Dexamethasone	NR3C 1	Homo sapiens	Dexamethasone binds to and results in increased activity of and results in increased localization of and results in increased degradation of NR3C1 protein
Dexamethasone	NR3C 1	Homo sapiens	Dexamethasone binds to and results in increased activity of NR3C1 protein
Dexamethasone	NR3C 1	Homo sapiens	[Dexamethasone binds to and results in increased activity of NR3C1 protein] which binds to SERPINA6 promoter
Dexamethasone	NR3C 1	Homo sapiens	[Dexamethasone binds to and results in increased activity of NR3C1 protein] which results in decreased expression of SERPINA6 mRNA
Dexamethasone	NR3C 1	Homo sapiens	Dexamethasone binds to NR3C1 protein
Dexamethasone	NR3C 1	Homo sapiens	[Dexamethasone binds to NR3C1 protein] which binds to CYP2C8 promoter
Dexamethasone	NR3C 1	Homo sapiens	[Dexamethasone binds to NR3C1 protein] which results in decreased expression of CCL2 protein
Dexamethasone	NR3C 1	Homo sapiens	[Dexamethasone co-treated with NCOA1 protein] results in increased activity of NR3C1 protein
Dexamethasone	NR3C 1	Homo sapiens	[Dexamethasone co-treated with NR3C1] results in decreased expression of HLA-DRA mRNA
Dexamethasone	NR3C 1	Homo sapiens	Dexamethasone inhibits the reaction [Tetrachlorodibenzodioxin results in decreased expression of NR3C1 protein]
Dexamethasone	NR3C 1	Homo sapiens	Dexamethasone promotes the reaction [Estradiol results in decreased expression of NR3C1 mRNA]
Dexamethasone	NR3C 1	Homo sapiens	Dexamethasone promotes the reaction [MAPK14 protein results in increased

			phosphorylation of NR3C1 protein]
Dexamethasone	NR3C 1	Homo sapiens	Dexamethasone promotes the reaction [NR3C1 protein affects the reaction [NR1I2 protein promotes the reaction [Clotrimazole results in increased expression of CYP2B6 mRNA]]]
Dexamethasone	NR3C 1	Homo sapiens	Dexamethasone promotes the reaction [NR3C1 protein affects the reaction [NR1I2 protein promotes the reaction [Phenobarbital results in increased expression of CYP2B6 mRNA]]]
Dexamethasone	NR3C 1	Homo sapiens	Dexamethasone promotes the reaction [NR3C1 protein affects the reaction [NR1I2 protein promotes the reaction [Phenytoin results in increased expression of CYP2B6 mRNA]]]
Dexamethasone	NR3C 1	Homo sapiens	Dexamethasone promotes the reaction [NR3C1 protein affects the reaction [NR1I2 protein promotes the reaction [Rifampin results in increased expression of CYP2B6 mRNA]]]
Dexamethasone	NR3C 1	Homo sapiens	Dexamethasone promotes the reaction [NR3C1 protein binds to CXCL8 promoter]
Dexamethasone	NR3C 1	Homo sapiens	Dexamethasone promotes the reaction [NR3C1 protein binds to CYP27A1 promoter]
Dexamethasone	NR3C 1	Homo sapiens	Dexamethasone promotes the reaction [NR3C1 protein binds to DKK1 promoter]
Dexamethasone	NR3C 1	Homo sapiens	Dexamethasone promotes the reaction [NR3C1 protein binds to GSTA1 promoter]
Dexamethasone	NR3C 1	Homo sapiens	Dexamethasone promotes the reaction [NR3C1 protein binds to TSC22D3 promoter]
Dexamethasone	NR3C 1	Homo sapiens	Dexamethasone results in decreased expression of NR3C1
Dexamethasone	NR3C 1	Homo sapiens	Dexamethasone results in decreased expression of NR3C1 mRNA
Dexamethasone	NR3C 1	Homo sapiens	Dexamethasone results in decreased expression of NR3C1 protein
Dexamethasone	NR3C 1	Homo sapiens	Dexamethasone results in increased activity of NR3C1 protein
Dexamethasone	NR3C 1	Homo sapiens	Dexamethasone results in increased expression of NR3C1 mRNA
Dexamethasone	NR3C 1	Homo sapiens	Dexamethasone results in increased localization of and results in increased activity of NR3C1 protein mutant form
Dexamethasone	NR3C 1	Homo sapiens	Dexamethasone results in increased localization of and results in increased phosphorylation of NR3C1 protein

Dexamethasone	NR3C 1	Homo sapiens	Dexamethasone results in increased localization of NR3C1 protein
Dexamethasone	NR3C 1	Homo sapiens	Dexamethasone results in increased phosphorylation of and results in increased activity of NR3C1 protein
Dexamethasone	NR3C 1	Homo sapiens	[Dexamethasone results in increased phosphorylation of and results in increased activity of NR3C1 protein] which results in increased expression of IGFBP1 mRNA
Dexamethasone	NR3C 1	Homo sapiens	[Dexamethasone results in increased phosphorylation of and results in increased activity of NR3C1 protein] which results in increased expression of IRF8 mRNA
Dexamethasone	NR3C 1	Homo sapiens	[Dexamethasone results in increased phosphorylation of and results in increased activity of NR3C1 protein] which results in increased expression of LAD1 mRNA
Dexamethasone	NR3C 1	Homo sapiens	[Dexamethasone results in increased phosphorylation of and results in increased activity of NR3C1 protein] which results in increased expression of TSC22D3 mRNA
Dexamethasone	NR3C 1	Homo sapiens	Dexamethasone results in increased phosphorylation of NR3C1 protein
Dexamethasone	NR3C 1	Homo sapiens	Dithiothreitol inhibits the reaction [arsenite inhibits the reaction [Dexamethasone binds to NR3C1 protein]]
Dexamethasone	NR3C 1	Homo sapiens	Dithiothreitol inhibits the reaction [Thiram inhibits the reaction [Dexamethasone binds to NR3C1 protein]]
Dexamethasone	NR3C 1	Homo sapiens	[Estradiol co-treated with Dexamethasone] inhibits the reaction [NR3C1 protein binds to TSC22D3 promoter]
Dexamethasone	NR3C 1	Homo sapiens	Genistein inhibits the reaction [Dexamethasone promotes the reaction [NR3C1 protein binds to TSC22D3 promoter]]
Dexamethasone	NR3C 1	Homo sapiens	inhibits the reaction [Dexamethasone binds to NR3C1 protein]
Dexamethasone	NR3C 1	Homo sapiens	Ketoconazole inhibits the reaction [Dexamethasone binds to and results in increased activity of NR3C1 protein]
Dexamethasone	NR3C 1	Homo sapiens	Melatonin inhibits the reaction [Dexamethasone results in increased activity of NR3C1 protein]
Dexamethasone	NR3C 1	Homo sapiens	Melatonin promotes the reaction [Dexamethasone results in decreased expression of NR3C1 mRNA]
Dexamethasone	NR3C	Homo	Miconazole inhibits the reaction

	1	sapiens	[Dexamethasone binds to and results in increased activity of NR3C1 protein]
Dexamethasone	NR3C 1	Homo sapiens	[Mifepristone binds to and results in decreased activity of NR3C1 protein] which results in decreased susceptibility to Dexamethasone
Dexamethasone	NR3C 1	Homo sapiens	Mifepristone inhibits the reaction [Dexamethasone binds to and results in increased activity of NR3C1 protein]
Dexamethasone	NR3C 1	Homo sapiens	Mifepristone inhibits the reaction [Dexamethasone results in decreased expression of NR3C1 mRNA]
Dexamethasone	NR3C 1	Homo sapiens	Mifepristone inhibits the reaction [NR3C1 protein promotes the reaction [Dexamethasone results in increased expression of CYP3A4 mRNA]]
Dexamethasone	NR3C 1	Homo sapiens	NR3C1 gene mutant form results in decreased susceptibility to Dexamethasone
Dexamethasone	NR3C 1	Homo sapiens	NR3C1 mutant form inhibits the reaction [Dexamethasone inhibits the reaction [Genistein results in increased expression of CA12 mRNA]]
Dexamethasone	NR3C 1	Homo sapiens	NR3C1 mutant form inhibits the reaction [Dexamethasone results in increased expression of LEFTY1 mRNA]
Dexamethasone	NR3C 1	Homo sapiens	NR3C1 mutant form inhibits the reaction [Dexamethasone results in increased expression of TSC22D3 mRNA]
Dexamethasone	NR3C 1	Homo sapiens	NR3C1 protein affects the reaction [Dexamethasone promotes the reaction [Methylcholanthrene results in increased expression of MT2A mRNA]]
Dexamethasone	NR3C 1	Homo sapiens	NR3C1 protein affects the reaction [Dexamethasone results in increased expression of and results in increased activity of HSD11B1 protein]
Dexamethasone	NR3C 1	Homo sapiens	NR3C1 protein affects the reaction [Dexamethasone results in increased expression of BCL2L11 protein]
Dexamethasone	NR3C 1	Homo sapiens	NR3C1 protein affects the reaction [Dexamethasone results in increased expression of CYP2A6 mRNA]
Dexamethasone	NR3C 1	Homo sapiens	NR3C1 protein affects the reaction [Dexamethasone results in increased expression of CYP3A5 mRNA]
Dexamethasone	NR3C 1	Homo sapiens	NR3C1 protein affects the reaction [Dexamethasone results in increased

			expression of MT2A mRNA]
Dexamethasone	NR3C 1	Homo sapiens	NR3C1 protein affects the reaction [Dexamethasone results in increased phosphorylation of PTK2B protein]
Dexamethasone	NR3C 1	Homo sapiens	NR3C1 protein affects the reaction [Methylcholanthrene promotes the reaction [Dexamethasone results in increased expression of MT2A mRNA]]
Dexamethasone	NR3C 1	Homo sapiens	NR3C1 protein affects the susceptibility to Dexamethasone
Dexamethasone	NR3C 1	Homo sapiens	NR3C1 protein alternative form inhibits the reaction [Dexamethasone results in increased activity of NR3C1 protein alternative form]
Dexamethasone	NR3C 1	Homo sapiens	[NR3C1 protein co-treated with Dexamethasone] inhibits the reaction [Methylcholanthrene results in increased expression of CYP1A1 mRNA]
Dexamethasone	NR3C 1	Homo sapiens	[NR3C1 protein co-treated with Dexamethasone] results in decreased susceptibility to Methylcholanthrene
Dexamethasone	NR3C 1	Homo sapiens	NR3C1 protein inhibits the reaction [Dexamethasone results in increased expression of DKK1 mRNA]
Dexamethasone	NR3C 1	Homo sapiens	NR3C1 protein promotes the reaction [Dexamethasone inhibits the reaction [TNF protein results in increased expression of IL6 mRNA]]
Dexamethasone	NR3C 1	Homo sapiens	NR3C1 protein promotes the reaction [Dexamethasone results in decreased expression of CYP2S1 mRNA]
Dexamethasone	NR3C 1	Homo sapiens	NR3C1 protein promotes the reaction [Dexamethasone results in decreased expression of CYP2S1 protein]
Dexamethasone	NR3C 1	Homo sapiens	NR3C1 protein promotes the reaction [Dexamethasone results in increased expression of CYP3A4 mRNA]
Dexamethasone	NR3C 1	Homo sapiens	NR3C1 protein promotes the reaction [Dexamethasone results in increased expression of G6PC2 mRNA]
Dexamethasone	NR3C 1	Homo sapiens	NR3C1 protein promotes the reaction [NR1I3 protein affects the reaction [[Dexamethasone co-treated with Phenobarbital] results in increased expression of CYP2B6 mRNA]]
Dexamethasone	NR3C 1	Homo sapiens	Paraquat inhibits the reaction [Dexamethasone results in increased activity of NR3C1 protein]
Dexamethasone	NR3C	Homo	Parathion inhibits the reaction

	1	sapiens	[Dexamethasone results in increased activity of NR3C1 protein]
Dexamethasone	NR3C 1	Homo sapiens	PCB 180 metabolite inhibits the reaction [Dexamethasone results in increased activity of NR3C1 protein]
Dexamethasone	NR3C 1	Homo sapiens	Pertussis Toxin inhibits the reaction [Melatonin inhibits the reaction [Dexamethasone results in increased activity of NR3C1 protein]]
Dexamethasone	NR3C 1	Homo sapiens	PPP2CA protein promotes the reaction [Dexamethasone results in increased localization of and results in increased phosphorylation of NR3C1 protein]
Dexamethasone	NR3C 1	Homo sapiens	SB 203580 inhibits the reaction [Dexamethasone results in increased phosphorylation of NR3C1 protein]
Dexamethasone	NR3C 1	Homo sapiens	Selenious Acid inhibits the reaction [Dexamethasone binds to NR3C1 protein]
Dexamethasone	NR3C 1	Homo sapiens	tetrabromobisphenol A inhibits the reaction [Dexamethasone results in increased activity of NR3C1 protein]
Dexamethasone	NR3C 1	Homo sapiens	[Tetrachlorodibenzodioxin co-treated with Dexamethasone] results in decreased expression of NR3C1
Dexamethasone	NR3C 1	Homo sapiens	Thiram inhibits the reaction [Dexamethasone binds to NR3C1 protein]
Dexamethasone	NR3C 1	Homo sapiens	Tolterodine Tartrate inhibits the reaction [Dexamethasone results in increased expression of NR3C1 mRNA]
Dexamethasone	NR3C 1	Homo sapiens	[Ursodeoxycholic Acid co-treated with Dexamethasone] promotes the reaction [EP300 protein binds to NR3C1 protein binds to HNF1A protein binds to SLC4A2 promoter alternative form]
Dexamethasone	NR3C 1	Homo sapiens	[Ursodeoxycholic Acid co-treated with Dexamethasone] promotes the reaction [HNF1A protein binds to NR3C1 protein binds to SLC4A2 promoter]
Dexamethasone	NR3C 1	Homo sapiens	[Ursodeoxycholic Acid co-treated with Dexamethasone] promotes the reaction [HNF1B protein binds to NR3C1 protein binds to EP300 protein binds to SLC4A2 promoter alternative form]
Dexamethasone	NR3C 1	Homo sapiens	[Ursodeoxycholic Acid co-treated with Dexamethasone] promotes the reaction [NR3C1 protein binds to SLC4A2 promoter alternative form]

Dexamethasone	NR3C 1	Homo sapiens	[Ursodeoxycholic Acid co-treated with Dexamethasone] results in increased localization of NR3C1 protein
Cisplatin	NR3C 1	Homo sapiens	Cisplatin results in decreased expression of NR3C1 mRNA
Dichlorodiphenyl Dichloroethylene	NR3C 1	Homo sapiens	Dichlorodiphenyl Dichloroethylene inhibits the reaction [Hydrocortisone results in increased activity of NR3C1 protein]
Dichlorodiphenyl Dichloroethylene	NR3C 1	Homo sapiens	Dichlorodiphenyl Dichloroethylene results in increased activity of NR3C1 protein
Diclofenac	NR3C 1	Homo sapiens	Diclofenac affects the expression of NR3C1 mRNA
Diethylhexyl Phthalate	NR3C 1	Homo sapiens	[Diethylhexyl Phthalate co-treated with butylparaben co-treated with propylparaben] results in increased activity of NR3C1 protein
Diethylhexyl Phthalate	NR3C 1	Homo sapiens	[Diethylhexyl Phthalate co-treated with butylparaben co-treated with tetramethrin co-treated with propylparaben] results in increased activity of NR3C1 protein
Diethylhexyl Phthalate	NR3C 1	Homo sapiens	[Diethylhexyl Phthalate co-treated with butylparaben co-treated with tetramethrin] results in increased activity of NR3C1 protein
Diethylhexyl Phthalate	NR3C 1	Homo sapiens	[Diethylhexyl Phthalate co-treated with butylparaben] results in increased activity of NR3C1 protein
Diethylhexyl Phthalate	NR3C 1	Homo sapiens	[Diethylhexyl Phthalate co-treated with propylparaben] results in increased activity of NR3C1 protein
Diethylhexyl Phthalate	NR3C 1	Homo sapiens	Diethylhexyl Phthalate results in increased activity of NR3C1 protein
Promegestone	NR3C 1	Homo sapiens	Promegestone results in increased phosphorylation of NR3C1 protein
Dinitrochlorobenzene	NR3C 1	Homo sapiens	Dinitrochlorobenzene results in increased expression of NR3C1 mRNA
Dithiothreitol	NR3C 1	Homo sapiens	Dithiothreitol inhibits the reaction [arsenite inhibits the reaction [Dexamethasone binds to NR3C1 protein]]
Dithiothreitol	NR3C 1	Homo sapiens	Dithiothreitol inhibits the reaction [Thiram inhibits the reaction [Dexamethasone binds to NR3C1 protein]]
Doxorubicin	NR3C 1	Homo sapiens	Doxorubicin results in decreased activity of NR3C1 protein
Dust	NR3C 1	Homo sapiens	Dust analog binds to and results in decreased activity of NR3C1 protein
Dust	NR3C 1	Homo sapiens	Dust analog binds to and results in increased activity of NR3C1 protein
E 3330	NR3C	Homo	E 3330 results in increased localization of

	NR3C1	Homo sapiens	NR3C1 protein
entinostat	NR3C1	Homo sapiens	entinostat results in increased expression of NR3C1 mRNA
entinostat	NR3C1	Homo sapiens	[NOG protein co-treated with entinostat co-treated with (6-(4-(2-piperidin-1-ylethoxy)phenyl))-3-pyridin-4-ylpyrazolo(1,5-a)pyrimidine co-treated with 4-(5-benzo(1,3)dioxol-5-yl-4-pyridin-2-yl-1H-imidazol-2-yl)benzamide] results in increased expression of NR3C1 mRNA
epigallocatechin gallate	NR3C1	Homo sapiens	epigallocatechin gallate results in decreased expression of NR3C1 mRNA
epigallocatechin gallate	NR3C1	Homo sapiens	[potassium chromate(VI) co-treated with epigallocatechin gallate] results in decreased expression of NR3C1 mRNA
ethyl myristate	NR3C1	Homo sapiens	ethyl myristate binds to and results in decreased activity of NR3C1 protein
Zearalenone	NR3C1	Homo sapiens	Zearalenone results in decreased expression of NR3C1 mRNA
fenchone	NR3C1	Homo sapiens	fenchone binds to and results in decreased activity of NR3C1 protein
Flame Retardants	NR3C1	Homo sapiens	Flame Retardants binds to and results in decreased activity of NR3C1 protein
Flumethasone	NR3C1	Homo sapiens	Flumethasone results in increased activity of NR3C1 protein
Fludrocortisone	NR3C1	Homo sapiens	Fludrocortisone binds to and results in increased activity of NR3C1 protein
Fluoxetine	NR3C1	Homo sapiens	Fluoxetine inhibits the reaction [IFNA1 protein results in decreased expression of NR3C1 mRNA]
Fluoxetine	NR3C1	Homo sapiens	Fluoxetine inhibits the reaction [IFNA1 protein results in decreased expression of NR3C1 protein]
Flutamide	NR3C1	Homo sapiens	Flutamide inhibits the reaction [Dehydroepiandrosterone results in increased expression of NR3C1 protein]
Fluticasone	NR3C1	Homo sapiens	Fluticasone affects the localization of and results in increased activity of NR3C1 protein
Fluticasone	NR3C1	Homo sapiens	Fluticasone affects the localization of NR3C1 protein
Fluticasone	NR3C1	Homo sapiens	Fluticasone binds to NR3C1 protein
Fluticasone	NR3C1	Homo sapiens	[Fluticasone co-treated with Salmeterol Xinafoate] affects the localization of and results in increased activity of NR3C1 protein
Fluticasone	NR3C1	Homo sapiens	[Fluticasone co-treated with Salmeterol Xinafoate] affects the localization of NR3C1

			protein
Fluticasone	NR3C1	Homo sapiens	[Fluticasone co-treated with Tetradecanoylphorbol Acetate] affects the localization of NR3C1 protein
Fluticasone	NR3C1	Homo sapiens	Fluticasone promotes the reaction [15-deoxyprostaglandin J2 promotes the reaction [NR3C1 protein binds to PPARG protein]]
Fluticasone	NR3C1	Homo sapiens	Salmeterol Xinafoate promotes the reaction [Fluticasone affects the localization of and results in increased activity of NR3C1 protein]
Fluticasone	NR3C1	Homo sapiens	Salmeterol Xinafoate promotes the reaction [Fluticasone affects the localization of NR3C1 protein]
Fluticasone	NR3C1	Homo sapiens	Salmeterol Xinafoate promotes the reaction [[Fluticasone co-treated with Tetradecanoylphorbol Acetate] affects the localization of NR3C1 protein]
Formaldehyde	NR3C1	Homo sapiens	Formaldehyde results in increased expression of NR3C1 mRNA
Formoterol Fumarate	NR3C1	Homo sapiens	[Beclomethasone co-treated with Formoterol Fumarate] affects the localization of NR3C1 protein
Genistein	NR3C1	Homo sapiens	Genistein inhibits the reaction [Dexamethasone promotes the reaction [NR3C1 protein binds to TSC22D3 promoter]]
Genistein	NR3C1	Homo sapiens	NR3C1 mutant form inhibits the reaction [Dexamethasone inhibits the reaction [Genistein results in increased expression of CA12 mRNA]]
ginsenoside Re	NR3C1	Homo sapiens	ginsenoside Re results in increased activity of NR3C1 protein
ginsenoside Re	NR3C1	Homo sapiens	[ginsenoside Re results in increased activity of NR3C1 protein] which results in increased abundance of Nitric Oxide
GKT137831	NR3C1	Homo sapiens	GKT137831 inhibits the reaction [anacetrapib results in increased expression of NR3C1 mRNA]
GKT137831	NR3C1	Homo sapiens	GKT137831 inhibits the reaction [torcetrapib results in increased expression of NR3C1 mRNA]
Glucocorticoids	NR3C1	Homo sapiens	Glucocorticoids results in increased expression of NR3C1 mRNA
Glucocorticoids	NR3C1	Homo sapiens	[HSD11B1 protein results in increased activity of Glucocorticoids] which results in increased activity of NR3C1 protein
Glucocorticoids	NR3C1	Homo sapiens	NR3C1 gene mutant form results in decreased susceptibility to Glucocorticoids

Glucocorticoids	NR3C1	Homo sapiens	NR3C1 gene polymorphism results in increased susceptibility to Glucocorticoids
Hydralazine	NR3C1	Homo sapiens	[Hydralazine co-treated with Valproic Acid] results in increased expression of NR3C1 mRNA
Hydrocortisone	NR3C1	Homo sapiens	2,2',4,4',5-brominated diphenyl ether inhibits the reaction [Hydrocortisone results in increased activity of NR3C1 protein]
Hydrocortisone	NR3C1	Homo sapiens	3-phenoxybenzoic acid inhibits the reaction [Hydrocortisone results in increased activity of NR3C1 protein]
Hydrocortisone	NR3C1	Homo sapiens	Atrazine inhibits the reaction [Hydrocortisone results in increased activity of NR3C1 protein]
Hydrocortisone	NR3C1	Homo sapiens	bifenthrin inhibits the reaction [Hydrocortisone results in increased activity of NR3C1 protein]
Hydrocortisone	NR3C1	Homo sapiens	croneton inhibits the reaction [Hydrocortisone results in increased activity of NR3C1 protein]
Hydrocortisone	NR3C1	Homo sapiens	cyhalothrin inhibits the reaction [Hydrocortisone results in increased activity of NR3C1 protein]
Hydrocortisone	NR3C1	Homo sapiens	cypermethrin inhibits the reaction [Hydrocortisone results in increased activity of NR3C1 protein]
Hydrocortisone	NR3C1	Homo sapiens	DDT inhibits the reaction [Hydrocortisone results in increased activity of NR3C1 protein]
Hydrocortisone	NR3C1	Homo sapiens	Dichlorodiphenyl Dichloroethylene inhibits the reaction [Hydrocortisone results in increased activity of NR3C1 protein]
Hydrocortisone	NR3C1	Homo sapiens	Hydrocortisone binds to and affects the activity of NR3C1 protein
Hydrocortisone	NR3C1	Homo sapiens	Hydrocortisone binds to and results in increased activity of NR3C1 protein
Hydrocortisone	NR3C1	Homo sapiens	Hydrocortisone binds to NR3C1 protein
Hydrocortisone	NR3C1	Homo sapiens	[Hydrocortisone co-treated with Progesterone] results in increased expression of NR3C1 mRNA
Hydrocortisone	NR3C1	Homo sapiens	Hydrocortisone results in decreased expression of NR3C1 mRNA
Hydrocortisone	NR3C1	Homo sapiens	Hydrocortisone results in decreased expression of NR3C1 protein
Hydrocortisone	NR3C1	Homo sapiens	Hydrocortisone results in increased activity of NR3C1 protein
Hydrocortisone	NR3C1	Homo sapiens	[Hydrocortisone results in increased activity of NR3C1 protein] inhibits the reaction [sulforafan results in increased activity of

			NFE2L2 protein]
Hydrocortisone	NR3C1	Homo sapiens	Hydrocortisone results in increased expression of NR3C1
Hydrocortisone	NR3C1	Homo sapiens	Hydrocortisone results in increased expression of NR3C1 mRNA
Hydrocortisone	NR3C1	Homo sapiens	Hydrocortisone results in increased phosphorylation of NR3C1 protein
Hydrocortisone	NR3C1	Homo sapiens	Methoxychlor inhibits the reaction [Hydrocortisone results in increased activity of NR3C1 protein]
Hydrocortisone	NR3C1	Homo sapiens	Mifepristone inhibits the reaction [Hydrocortisone results in increased activity of NR3C1 protein]
Hydrocortisone	NR3C1	Homo sapiens	N-dichlorofluoromethylthio-N',N'-dimethyl-N-p-tolylsulfamide inhibits the reaction [Hydrocortisone results in increased activity of NR3C1 protein]
Hydrocortisone	NR3C1	Homo sapiens	NR3C1 gene polymorphism affects the abundance of Hydrocortisone
Hydrocortisone	NR3C1	Homo sapiens	NR3C1 protein alternative form inhibits the reaction [Hydrocortisone results in increased activity of NR3C1 protein alternative form]
Hydrocortisone	NR3C1	Homo sapiens	NR3C1 protein promotes the reaction [Hydrocortisone inhibits the reaction [IL1A protein results in increased activity of MMP9 protein]]
Hydrocortisone	NR3C1	Homo sapiens	o,p'-DDT inhibits the reaction [Hydrocortisone results in increased activity of NR3C1 protein]
Hydrocortisone	NR3C1	Homo sapiens	pentabrominated diphenyl ether 100 inhibits the reaction [Hydrocortisone results in increased activity of NR3C1 protein]
Hydrocortisone	NR3C1	Homo sapiens	Polybrominated Biphenyls metabolite inhibits the reaction [Hydrocortisone results in increased activity of NR3C1 protein]
Hydrocortisone	NR3C1	Homo sapiens	resmethrin inhibits the reaction [Hydrocortisone results in increased activity of NR3C1 protein]
Hydrocortisone	NR3C1	Homo sapiens	Thiram inhibits the reaction [Hydrocortisone binds to NR3C1 protein]
Hydrocortisone	NR3C1	Homo sapiens	tributyl phosphate inhibits the reaction [Hydrocortisone results in increased activity of NR3C1 protein]
Hydrocortisone	NR3C1	Homo sapiens	triphenyl phosphate inhibits the reaction [Hydrocortisone results in increased activity of NR3C1 protein]
Hydrocortisone	NR3C1	Homo sapiens	triphenyl phosphate metabolite inhibits the reaction [Hydrocortisone results in increased

			activity of NR3C1 protein]
Hydrocortisone	NR3C 1	Homo sapiens	tris(1,3-dichloro-2-propyl)phosphate inhibits the reaction [Hydrocortisone results in increased activity of NR3C1 protein]
Hydrogen Peroxide	NR3C 1	Homo sapiens	Hydrogen Peroxide affects the expression of NR3C1 mRNA
17-alpha-Hydroxyprogesterone	NR3C 1	Homo sapiens	17-alpha-Hydroxyprogesterone analog binds to and results in increased activity of NR3C1 protein
Desoxycorticosterone	NR3C 1	Homo sapiens	Desoxycorticosterone binds to and results in increased activity of NR3C1 protein
Desoxycorticosterone	NR3C 1	Homo sapiens	Desoxycorticosterone results in increased phosphorylation of NR3C1 protein
Ibuprofen	NR3C 1	Homo sapiens	Ibuprofen results in decreased expression of NR3C1 mRNA
irinotecan	NR3C 1	Homo sapiens	NR3C1 protein results in decreased susceptibility to irinotecan
Isoflurophate	NR3C 1	Homo sapiens	Isoflurophate results in decreased degradation of NR3C1 protein
Isoquinolines	NR3C 1	Homo sapiens	Isoquinolines analog binds to and results in decreased activity of NR3C1 protein
jinfukang	NR3C 1	Homo sapiens	jinfukang results in decreased expression of NR3C1 mRNA
K 7174	NR3C 1	Homo sapiens	K 7174 results in increased expression of NR3C1 mRNA
Ketoconazole	NR3C 1	Homo sapiens	Ketoconazole inhibits the reaction [Dexamethasone binds to and results in increased activity of NR3C1 protein]
Ketoconazole	NR3C 1	Homo sapiens	Ketoconazole results in decreased activity of NR3C1 protein
lamotrigine	NR3C 1	Homo sapiens	NR3C1 protein results in increased susceptibility to lamotrigine
laurolactam	NR3C 1	Homo sapiens	laurolactam binds to and results in decreased activity of NR3C1 protein
leflunomide	NR3C 1	Homo sapiens	leflunomide results in increased expression of NR3C1 mRNA
Medroxyprogesterone Acetate	NR3C 1	Homo sapiens	[Medroxyprogesterone Acetate co-treated with 8-Bromo Cyclic Adenosine Monophosphate] results in decreased expression of NR3C1 mRNA
Medroxyprogesterone Acetate	NR3C 1	Homo sapiens	[Medroxyprogesterone Acetate co-treated with 8-Bromo Cyclic Adenosine Monophosphate] results in decreased expression of NR3C1 protein
Medroxyprogesterone Acetate	NR3C 1	Homo sapiens	NR3C1 protein affects the susceptibility to Medroxyprogesterone Acetate
Melatonin	NR3C	Homo	Melatonin inhibits the reaction

	1	sapiens	[Dexamethasone results in increased activity of NR3C1 protein]
Melatonin	NR3C 1	Homo sapiens	Melatonin promotes the reaction [Dexamethasone results in decreased expression of NR3C1 mRNA]
Melatonin	NR3C 1	Homo sapiens	Pertussis Toxin inhibits the reaction [Melatonin inhibits the reaction [Dexamethasone results in increased activity of NR3C1 protein]]
mercuric bromide	NR3C 1	Homo sapiens	mercuric bromide results in increased expression of NR3C1 mRNA
mercuric bromide	NR3C 1	Homo sapiens	[NOG protein co-treated with mercuric bromide co-treated with (6-(4-(2-piperidin-1-ylethoxy)phenyl))-3-pyridin-4-ylpyrazolo(1,5-a)pyrimidine co-treated with 4-(5-benzo(1,3)dioxol-5-yl-4-pyridin-2-yl-1H-imidazol-2-yl)benzamide] results in increased expression of NR3C1 mRNA
Metformin	NR3C 1	Homo sapiens	Metformin results in decreased expression of NR3C1 mRNA
Metformin	NR3C 1	Homo sapiens	Metformin results in decreased expression of NR3C1 protein
Methotrexate	NR3C 1	Homo sapiens	Methotrexate results in decreased expression of NR3C1 mRNA
Methoxychlor	NR3C 1	Homo sapiens	Methoxychlor inhibits the reaction [Hydrocortisone results in increased activity of NR3C1 protein]
Methylcholanthrene	NR3C 1	Homo sapiens	NR3C1 protein affects the reaction [Dexamethasone promotes the reaction [Methylcholanthrene results in increased expression of MT2A mRNA]]
Methylcholanthrene	NR3C 1	Homo sapiens	NR3C1 protein affects the reaction [Methylcholanthrene promotes the reaction [Dexamethasone results in increased expression of MT2A mRNA]]
Methylcholanthrene	NR3C 1	Homo sapiens	NR3C1 protein affects the reaction [Methylcholanthrene results in increased expression of MT2A mRNA]
Methylcholanthrene	NR3C 1	Homo sapiens	[NR3C1 protein co-treated with Dexamethasone] inhibits the reaction [Methylcholanthrene results in increased expression of CYP1A1 mRNA]
Methylcholanthrene	NR3C 1	Homo sapiens	[NR3C1 protein co-treated with Dexamethasone] results in decreased susceptibility to Methylcholanthrene
methylmercuric chloride	NR3C 1	Homo sapiens	methylmercuric chloride results in increased expression of NR3C1 mRNA

1-Methyl-4-phenylpyridinium	NR3C1	Homo sapiens	1-Methyl-4-phenylpyridinium results in increased expression of NR3C1 mRNA
methylselenic acid	NR3C1	Homo sapiens	methylselenic acid affects the expression of NR3C1 mRNA
Miconazole	NR3C1	Homo sapiens	Miconazole inhibits the reaction [Dexamethasone binds to and results in increased activity of NR3C1 protein]
Mifepristone	NR3C1	Homo sapiens	Mifepristone analog binds to and results in decreased activity of NR3C1 protein
Mifepristone	NR3C1	Homo sapiens	Mifepristone binds to and results in decreased activity of NR3C1 protein
Mifepristone	NR3C1	Homo sapiens	[Mifepristone binds to and results in decreased activity of NR3C1 protein] inhibits the reaction [[HSD11B1 protein results in increased activity of Cortisone] inhibits the reaction [sulforafan results in increased activity of NFE2L2 protein]]
Mifepristone	NR3C1	Homo sapiens	[Mifepristone binds to and results in decreased activity of NR3C1 protein] which results in decreased susceptibility to Dexamethasone
Mifepristone	NR3C1	Homo sapiens	Mifepristone binds to NR3C1 protein
Mifepristone	NR3C1	Homo sapiens	Mifepristone inhibits the reaction [Dexamethasone binds to and results in increased activity of NR3C1 protein]
Mifepristone	NR3C1	Homo sapiens	Mifepristone inhibits the reaction [Dexamethasone results in decreased expression of NR3C1 mRNA]
Mifepristone	NR3C1	Homo sapiens	Mifepristone inhibits the reaction [Hydrocortisone results in increased activity of NR3C1 protein]
Mifepristone	NR3C1	Homo sapiens	Mifepristone inhibits the reaction [NR3C1 protein promotes the reaction [Dexamethasone results in increased expression of CYP3A4 mRNA]]
Mifepristone	NR3C1	Homo sapiens	Mifepristone results in decreased expression of NR3C1 mRNA
Mifepristone	NR3C1	Homo sapiens	Mifepristone results in decreased expression of NR3C1 protein
Mifepristone	NR3C1	Homo sapiens	Mifepristone results in increased activity of NR3C1 protein
Mifepristone	NR3C1	Homo sapiens	Mifepristone results in increased expression of NR3C1 mRNA
Mitoxantrone	NR3C1	Homo sapiens	Mitoxantrone results in decreased activity of NR3C1 protein
N-butylbenzenesulfonamide	NR3C	Homo	N-butylbenzenesulfonamide binds to and

	1	sapiens	results in decreased activity of NR3C1 protein
N-dichlorofluoromethylthio-N',N'-dimethyl-N-p-tolylsulfamide	NR3C 1	Homo sapiens	N-dichlorofluoromethylthio-N',N'-dimethyl-N-p-tolylsulfamide inhibits the reaction [Hydrocortisone results in increased activity of NR3C1 protein]
nickel chloride	NR3C 1	Homo sapiens	nickel chloride results in increased expression of NR3C1 mRNA
nickel sulfate	NR3C 1	Homo sapiens	nickel sulfate results in increased expression of NR3C1 mRNA
Nitric Oxide	NR3C 1	Homo sapiens	[ginsenoside Re results in increased activity of NR3C1 protein] which results in increased abundance of Nitric Oxide
ON 01910	NR3C 1	Homo sapiens	ON 01910 results in increased expression of NR3C1 mRNA
onapristone	NR3C 1	Homo sapiens	onapristone binds to NR3C1 protein
o,p'-DDT	NR3C 1	Homo sapiens	o,p'-DDT inhibits the reaction [Hydrocortisone results in increased activity of NR3C1 protein]
Oxazolone	NR3C 1	Homo sapiens	Oxazolone results in increased expression of NR3C1 mRNA
Oxygen	NR3C 1	Homo sapiens	Oxygen deficiency results in increased expression of NR3C1 mRNA
Paraquat	NR3C 1	Homo sapiens	Paraquat inhibits the reaction [Dexamethasone results in increased activity of NR3C1 protein]
Paraquat	NR3C 1	Homo sapiens	Paraquat results in decreased activity of NR3C1 protein
pararosaniline	NR3C 1	Homo sapiens	pararosaniline results in decreased activity of NR3C1 protein
pararosaniline	NR3C 1	Homo sapiens	pararosaniline results in decreased expression of NR3C1 protein
Parathion	NR3C 1	Homo sapiens	Parathion inhibits the reaction [Dexamethasone results in increased activity of NR3C1 protein]
Parathion	NR3C 1	Homo sapiens	Parathion results in decreased activity of NR3C1 protein
PCB 180	NR3C 1	Homo sapiens	PCB 180 metabolite inhibits the reaction [Dexamethasone results in increased activity of NR3C1 protein]
pentabrominated diphenyl ether 100	NR3C 1	Homo sapiens	pentabrominated diphenyl ether 100 inhibits the reaction [Hydrocortisone results in increased activity of NR3C1 protein]
Pertussis Toxin	NR3C 1	Homo sapiens	Pertussis Toxin inhibits the reaction [Melatonin inhibits the reaction [Dexamethasone results in increased activity of NR3C1 protein]]
Phenobarbital	NR3C	Homo	Dexamethasone promotes the reaction

	1	sapiens	[NR3C1 protein affects the reaction [NR1I2 protein promotes the reaction [Phenobarbital results in increased expression of CYP2B6 mRNA]]]
Phenobarbital	NR3C1	Homo sapiens	NR3C1 protein promotes the reaction [NR1I3 protein affects the reaction [[Dexamethasone co-treated with Phenobarbital] results in increased expression of CYP2B6 mRNA]]
Phenobarbital	NR3C1	Homo sapiens	NR3C1 protein promotes the reaction [Phenobarbital results in increased expression of CYP3A4 mRNA]
Phenylbutazone	NR3C1	Homo sapiens	NR3C1 protein promotes the reaction [Phenylbutazone results in increased expression of CYP3A4 mRNA]
Phenylmercuric Acetate	NR3C1	Homo sapiens	[NOG protein co-treated with Phenylmercuric Acetate co-treated with (6-(4-(2-piperidin-1-ylethoxy)phenyl))-3-pyridin-4-ylpyrazolo(1,5-a)pyrimidine co-treated with 4-(5-benzo(1,3)dioxol-5-yl-4-pyridin-2-yl-1H-imidazol-2-yl)benzamide] results in increased expression of NR3C1 mRNA
Phenylmercuric Acetate	NR3C1	Homo sapiens	Phenylmercuric Acetate results in increased expression of NR3C1 mRNA
Phenytoin	NR3C1	Homo sapiens	Dexamethasone promotes the reaction [NR3C1 protein affects the reaction [NR1I2 protein promotes the reaction [Phenytoin results in increased expression of CYP2B6 mRNA]]]
Phenytoin	NR3C1	Homo sapiens	NR3C1 protein promotes the reaction [Phenytoin results in increased expression of CYP3A4 mRNA]
Plant Extracts	NR3C1	Homo sapiens	NR3C1 protein promotes the reaction [Plant Extracts inhibits the reaction [TNF protein results in increased expression of IL6 mRNA]]
Plant Extracts	NR3C1	Homo sapiens	Plant Extracts results in increased expression of NR3C1 mRNA
Polybrominated Biphenyls	NR3C1	Homo sapiens	Polybrominated Biphenyls metabolite inhibits the reaction [Hydrocortisone results in increased activity of NR3C1 protein]
potassium chromate(VI)	NR3C1	Homo sapiens	[potassium chromate(VI) co-treated with epigallocatechin gallate] results in decreased expression of NR3C1 mRNA
potassium chromate(VI)	NR3C1	Homo sapiens	potassium chromate(VI) results in decreased expression of NR3C1 mRNA
Prednisolone	NR3C1	Homo sapiens	NR3C1 protein alternative form affects the susceptibility to Prednisolone
Prednisolone	NR3C	Homo	NR3C1 protein alternative form inhibits the

	1	sapiens	reaction [Prednisolone results in increased activity of NR3C1 protein alternative form]
Prednisolone	NR3C1	Homo sapiens	Prednisolone binds to and results in increased activity of NR3C1 protein
Prednisolone	NR3C1	Homo sapiens	Prednisolone binds to NR3C1 protein
Prednisolone	NR3C1	Homo sapiens	Prednisolone results in increased activity of NR3C1 protein
prednylidene	NR3C1	Homo sapiens	prednylidene results in increased activity of NR3C1 protein
Pregnenolone Carbonitrile	NR3C1	Homo sapiens	NR3C1 protein promotes the reaction [Pregnenolone Carbonitrile results in increased expression of CYP3A4 mRNA]
Progesterone	NR3C1	Homo sapiens	[Hydrocortisone co-treated with Progesterone] results in increased expression of NR3C1 mRNA
Progesterone	NR3C1	Homo sapiens	NR3C1 protein inhibits the reaction [[Progesterone co-treated with 8-Bromo Cyclic Adenosine Monophosphate co-treated with Estradiol] results in decreased expression of GRIA1 mRNA]
Progesterone	NR3C1	Homo sapiens	NR3C1 protein inhibits the reaction [[Progesterone co-treated with 8-Bromo Cyclic Adenosine Monophosphate co-treated with Estradiol] results in increased expression of ZNF486 mRNA]
Progesterone	NR3C1	Homo sapiens	NR3C1 protein promotes the reaction [[Progesterone co-treated with 8-Bromo Cyclic Adenosine Monophosphate co-treated with Estradiol] results in increased expression of WNT4 mRNA]
Progesterone	NR3C1	Homo sapiens	Progesterone binds to and results in increased activity of NR3C1 protein
Progesterone	NR3C1	Homo sapiens	Progesterone binds to NR3C1 protein
Progesterone	NR3C1	Homo sapiens	[Progesterone co-treated with 8-Bromo Cyclic Adenosine Monophosphate co-treated with Estradiol] results in decreased expression of NR3C1 protein
Progesterone	NR3C1	Homo sapiens	[Progesterone co-treated with 8-Bromo Cyclic Adenosine Monophosphate] results in decreased expression of NR3C1 mRNA
Progesterone	NR3C1	Homo sapiens	Progesterone results in increased expression of NR3C1 mRNA
Progesterone	NR3C1	Homo sapiens	Progesterone results in increased phosphorylation of NR3C1 protein
propylparaben	NR3C	Homo	[butylparaben co-treated with propylparaben]

	1	sapiens	results in increased activity of NR3C1 protein
propylparaben	NR3C 1	Homo sapiens	[Diethylhexyl Phthalate co-treated with butylparaben co-treated with propylparaben] results in increased activity of NR3C1 protein
propylparaben	NR3C 1	Homo sapiens	[Diethylhexyl Phthalate co-treated with butylparaben co-treated with tetramethrin co-treated with propylparaben] results in increased activity of NR3C1 protein
propylparaben	NR3C 1	Homo sapiens	[Diethylhexyl Phthalate co-treated with propylparaben] results in increased activity of NR3C1 protein
propylparaben	NR3C 1	Homo sapiens	propylparaben results in increased activity of NR3C1 protein
propylparaben	NR3C 1	Homo sapiens	[tetramethrin co-treated with propylparaben] results in increased activity of NR3C1 protein
protoporphyrin IX	NR3C 1	Homo sapiens	protoporphyrin IX results in decreased expression of NR3C1 protein
Quercetin	NR3C 1	Homo sapiens	Quercetin results in decreased expression of NR3C1
Quercetin	NR3C 1	Homo sapiens	Quercetin results in increased expression of NR3C1 mRNA
Raloxifene Hydrochloride	NR3C 1	Homo sapiens	[Raloxifene Hydrochloride co-treated with ESR2 protein] results in increased expression of NR3C1 mRNA
Reactive Oxygen Species	NR3C 1	Homo sapiens	Reactive Oxygen Species affects the expression of NR3C1 mRNA
resmethrin	NR3C 1	Homo sapiens	resmethrin inhibits the reaction [Hydrocortisone results in increased activity of NR3C1 protein]
resveratrol	NR3C 1	Homo sapiens	[Coumestrol co-treated with resveratrol] results in decreased expression of NR3C1 mRNA
resveratrol	NR3C 1	Homo sapiens	resveratrol results in decreased expression of NR3C1 mRNA
resveratrol	NR3C 1	Homo sapiens	resveratrol results in decreased expression of NR3C1 protein
Rifampin	NR3C 1	Homo sapiens	Dexamethasone promotes the reaction [NR3C1 protein affects the reaction [NR1I2 protein promotes the reaction [Rifampin results in increased expression of CYP2B6 mRNA]]]
Rifampin	NR3C 1	Homo sapiens	NR3C1 protein promotes the reaction [Rifampin results in increased expression of CYP3A4 mRNA]
Rifampin	NR3C 1	Homo sapiens	Rifampin results in increased expression of NR3C1 mRNA
Rotenone	NR3C	Homo	Rotenone inhibits the reaction [torcetrapib

	1	sapiens	results in increased expression of NR3C1 mRNA]
Salmeterol Xinafoate	NR3C 1	Homo sapiens	[Fluticasone co-treated with Salmeterol Xinafoate] affects the localization of and results in increased activity of NR3C1 protein
Salmeterol Xinafoate	NR3C 1	Homo sapiens	[Fluticasone co-treated with Salmeterol Xinafoate] affects the localization of NR3C1 protein
Salmeterol Xinafoate	NR3C 1	Homo sapiens	Salmeterol Xinafoate affects the localization of and results in increased activity of NR3C1 protein
Salmeterol Xinafoate	NR3C 1	Homo sapiens	Salmeterol Xinafoate affects the localization of NR3C1 protein
Salmeterol Xinafoate	NR3C 1	Homo sapiens	Salmeterol Xinafoate promotes the reaction [15-deoxyprostaglandin J2 promotes the reaction [NR3C1 protein binds to PPARG protein]]
Salmeterol Xinafoate	NR3C 1	Homo sapiens	Salmeterol Xinafoate promotes the reaction [Fluticasone affects the localization of and results in increased activity of NR3C1 protein]
Salmeterol Xinafoate	NR3C 1	Homo sapiens	Salmeterol Xinafoate promotes the reaction [Fluticasone affects the localization of NR3C1 protein]
Salmeterol Xinafoate	NR3C 1	Homo sapiens	Salmeterol Xinafoate promotes the reaction [[Fluticasone co-treated with Tetradecanoylphorbol Acetate] affects the localization of NR3C1 protein]
SB 203580	NR3C 1	Homo sapiens	SB 203580 inhibits the reaction [deacylcortivazol results in increased phosphorylation of NR3C1 protein]
SB 203580	NR3C 1	Homo sapiens	SB 203580 inhibits the reaction [Dexamethasone results in increased phosphorylation of NR3C1 protein]
Selenious Acid	NR3C 1	Homo sapiens	Selenious Acid inhibits the reaction [Dexamethasone binds to NR3C1 protein]
Selenium	NR3C 1	Homo sapiens	Selenium results in decreased expression of NR3C1 mRNA
sodium arsenite	NR3C 1	Homo sapiens	sodium arsenite affects the activity of NR3C1 protein
sodium arsenite	NR3C 1	Homo sapiens	sodium arsenite affects the methylation of NR3C1 gene
Sodium Selenite	NR3C 1	Homo sapiens	Sodium Selenite affects the expression of NR3C1 mRNA
Soot	NR3C 1	Homo sapiens	Soot results in decreased expression of NR3C1 mRNA
spiroxamine	NR3C 1	Homo sapiens	spiroxamine results in increased activity of NR3C1 protein

Sulfasalazine	NR3C 1	Homo sapiens	Sulfasalazine results in increased expression of NR3C1 mRNA
Sulfasalazine	NR3C 1	Homo sapiens	Sulfasalazine results in increased expression of NR3C1 protein
Sulfonamides	NR3C 1	Homo sapiens	Sulfonamides analog binds to and results in decreased activity of NR3C1 protein
sulforafan	NR3C 1	Homo sapiens	[Hydrocortisone results in increased activity of NR3C1 protein] inhibits the reaction [sulforafan results in increased activity of NFE2L2 protein]
sulforafan	NR3C 1	Homo sapiens	[Mifepristone binds to and results in decreased activity of NR3C1 protein] inhibits the reaction [[HSD11B1 protein results in increased activity of Cortisone] inhibits the reaction [sulforafan results in increased activity of NFE2L2 protein]]
Tamoxifen	NR3C 1	Homo sapiens	[Tamoxifen co-treated with ESR2 protein] results in increased expression of NR3C1 mRNA
tanshinone	NR3C 1	Homo sapiens	NR3C1 protein affects the reaction [tanshinone results in increased expression of CYP3A4 protein]
tauroursodeoxycholic acid	NR3C 1	Homo sapiens	tauroursodeoxycholic acid inhibits the reaction [IFNA1 protein results in decreased expression of NR3C1 mRNA]
tauroursodeoxycholic acid	NR3C 1	Homo sapiens	tauroursodeoxycholic acid inhibits the reaction [IFNA1 protein results in decreased expression of NR3C1 protein]
Tenofovir	NR3C 1	Homo sapiens	Tenofovir affects the expression of NR3C1 mRNA
Terbutaline	NR3C 1	Homo sapiens	Budesonide affects the reaction [Terbutaline results in increased expression of NR3C1 mRNA]
Terbutaline	NR3C 1	Homo sapiens	Terbutaline results in increased expression of NR3C1 mRNA
testosterone-3-carboxymethyloxime-bovine serum albumin conjugate	NR3C 1	Homo sapiens	testosterone-3-carboxymethyloxime-bovine serum albumin conjugate results in decreased expression of NR3C1 mRNA
tetrabromobisphenol A	NR3C 1	Homo sapiens	tetrabromobisphenol A inhibits the reaction [Dexamethasone results in increased activity of NR3C1 protein]
Tetrachlorodibenzodioxin	NR3C 1	Homo sapiens	Dexamethasone inhibits the reaction [Tetrachlorodibenzodioxin results in decreased expression of NR3C1 protein]
Tetrachlorodibenzodioxin	NR3C 1	Homo sapiens	[Tetrachlorodibenzodioxin co-treated with Dexamethasone] results in decreased expression of NR3C1

Tetrachlorodibenzodioxin	NR3C 1	Homo sapiens	Tetrachlorodibenzodioxin promotes the reaction [AHR protein binds to FOXA1 protein binds to NR3C1 protein]
Tetrachlorodibenzodioxin	NR3C 1	Homo sapiens	Tetrachlorodibenzodioxin promotes the reaction [AHR protein binds to NR3C1 protein]
Tetrachlorodibenzodioxin	NR3C 1	Homo sapiens	Tetrachlorodibenzodioxin promotes the reaction [FOXA1 protein binds to NR3C1 protein]
Tetrachlorodibenzodioxin	NR3C 1	Homo sapiens	Tetrachlorodibenzodioxin results in decreased expression of NR3C1
Tetrachlorodibenzodioxin	NR3C 1	Homo sapiens	Tetrachlorodibenzodioxin results in increased expression of NR3C1 mRNA
Tetrachlorodibenzodioxin	NR3C 1	Homo sapiens	Tetrachlorodibenzodioxin results in increased expression of NR3C1 protein
Tetradecanoylphorbol Acetate	NR3C 1	Homo sapiens	[Fluticasone co-treated with Tetradecanoylphorbol Acetate] affects the localization of NR3C1 protein
Tetradecanoylphorbol Acetate	NR3C 1	Homo sapiens	Salmeterol Xinafoate promotes the reaction [[Fluticasone co-treated with Tetradecanoylphorbol Acetate] affects the localization of NR3C1 protein]
tetramethrin	NR3C 1	Homo sapiens	[Diethylhexyl Phthalate co-treated with butylparaben co-treated with tetramethrin co-treated with propylparaben] results in increased activity of NR3C1 protein
tetramethrin	NR3C 1	Homo sapiens	[Diethylhexyl Phthalate co-treated with butylparaben co-treated with tetramethrin] results in increased activity of NR3C1 protein
tetramethrin	NR3C 1	Homo sapiens	[tetramethrin co-treated with propylparaben] results in increased activity of NR3C1 protein
Thiram	NR3C 1	Homo sapiens	Dithiothreitol inhibits the reaction [Thiram inhibits the reaction [Dexamethasone binds to NR3C1 protein]]
Thiram	NR3C 1	Homo sapiens	Thiram inhibits the reaction [Dexamethasone binds to NR3C1 protein]
Thiram	NR3C 1	Homo sapiens	Thiram inhibits the reaction [Hydrocortisone binds to NR3C1 protein]
Tobacco Smoke Pollution	NR3C 1	Homo sapiens	Tobacco Smoke Pollution results in decreased methylation of NR3C1 promoter
Tolterodine Tartrate	NR3C 1	Homo sapiens	Tolterodine Tartrate inhibits the reaction [Dexamethasone results in increased expression of NR3C1 mRNA]
Tolterodine Tartrate	NR3C 1	Homo sapiens	Tolterodine Tartrate results in decreased expression of NR3C1 mRNA
torcetrapib	NR3C 1	Homo sapiens	2-acetylphenothiazine inhibits the reaction [torcetrapib results in increased expression of NR3C1 mRNA]

torcetrapib	NR3C1	Homo sapiens	Acetylcysteine inhibits the reaction [torcetrapib results in increased expression of NR3C1 mRNA]
torcetrapib	NR3C1	Homo sapiens	GKT137831 inhibits the reaction [torcetrapib results in increased expression of NR3C1 mRNA]
torcetrapib	NR3C1	Homo sapiens	Rotenone inhibits the reaction [torcetrapib results in increased expression of NR3C1 mRNA]
torcetrapib	NR3C1	Homo sapiens	torcetrapib results in increased expression of NR3C1 mRNA
Tretinoin	NR3C1	Homo sapiens	[Tretinoin co-treated with Ascorbic Acid] results in increased expression of NR3C1 mRNA
Tretinoin	NR3C1	Homo sapiens	Tretinoin results in increased expression of NR3C1 mRNA
Triamcinolone	NR3C1	Homo sapiens	NR3C1 protein alternative form inhibits the reaction [Triamcinolone results in increased activity of NR3C1 protein alternative form]
Triamcinolone	NR3C1	Homo sapiens	Triamcinolone binds to and results in increased activity of NR3C1 protein
Triamcinolone	NR3C1	Homo sapiens	Triamcinolone results in increased activity of NR3C1 protein
Triamcinolone Acetonide	NR3C1	Homo sapiens	Triamcinolone Acetonide binds to and affects the localization of NR3C1 protein
Triamcinolone Acetonide	NR3C1	Homo sapiens	Triamcinolone Acetonide binds to NR3C1 protein
Triamcinolone Acetonide	NR3C1	Homo sapiens	[Triamcinolone Acetonide binds to NR3C1 protein] which results in decreased secretion of VEGFA protein
Triamcinolone Acetonide	NR3C1	Homo sapiens	Triamcinolone Acetonide results in decreased expression of NR3C1 protein
tributyl phosphate	NR3C1	Homo sapiens	tributyl phosphate inhibits the reaction [Hydrocortisone results in increased activity of NR3C1 protein]
tributyl phosphate	NR3C1	Homo sapiens	tributyl phosphate results in decreased activity of NR3C1 protein
trichostatin A	NR3C1	Homo sapiens	trichostatin A affects the expression of NR3C1 mRNA
trichostatin A	NR3C1	Homo sapiens	trichostatin A results in decreased expression of NR3C1 mRNA alternative form
trichostatin A	NR3C1	Homo sapiens	trichostatin A results in decreased expression of NR3C1 protein alternative form
trichostatin A	NR3C1	Homo sapiens	trichostatin A results in increased expression of NR3C1 mRNA
trichostatin A	NR3C1	Homo sapiens	trichostatin A results in increased expression of NR3C1 mRNA alternative form

trichostatin A	NR3C1	Homo sapiens	trichostatin A results in increased expression of NR3C1 protein alternative form
triphenyl phosphate	NR3C1	Homo sapiens	triphenyl phosphate inhibits the reaction [Hydrocortisone results in increased activity of NR3C1 protein]
triphenyl phosphate	NR3C1	Homo sapiens	triphenyl phosphate metabolite inhibits the reaction [Hydrocortisone results in increased activity of NR3C1 protein]
triphenyl phosphate	NR3C1	Homo sapiens	triphenyl phosphate results in decreased activity of NR3C1 protein
triptolide	NR3C1	Homo sapiens	triptolide results in increased expression of and results in increased phosphorylation of NR3C1 protein
triptolide	NR3C1	Homo sapiens	triptolide results in increased expression of NR3C1 mRNA
tris(1,3-dichloro-2-propyl)phosphate	NR3C1	Homo sapiens	tris(1,3-dichloro-2-propyl)phosphate inhibits the reaction [Hydrocortisone results in increased activity of NR3C1 protein]
tris(1,3-dichloro-2-propyl)phosphate	NR3C1	Homo sapiens	tris(1,3-dichloro-2-propyl)phosphate results in decreased activity of NR3C1 protein
tris(2-ethylhexyl)phosphate	NR3C1	Homo sapiens	tris(2-ethylhexyl)phosphate binds to and results in decreased activity of NR3C1 protein
tris(2-ethylhexyl)phosphate	NR3C1	Homo sapiens	tris(2-ethylhexyl)phosphate results in decreased activity of NR3C1 protein
Tritolyl Phosphates	NR3C1	Homo sapiens	Tritolyl Phosphates results in decreased activity of NR3C1 protein
troglitazone	NR3C1	Homo sapiens	troglitazone results in decreased expression of NR3C1 mRNA
undecan-2-one	NR3C1	Homo sapiens	undecan-2-one binds to and results in decreased activity of NR3C1 protein
Urethane	NR3C1	Homo sapiens	Urethane results in decreased expression of NR3C1 mRNA
Ursodeoxycholic Acid	NR3C1	Homo sapiens	Ursodeoxycholic Acid affects the localization of NR3C1 protein
Ursodeoxycholic Acid	NR3C1	Homo sapiens	[Ursodeoxycholic Acid co-treated with Dexamethasone] promotes the reaction [EP300 protein binds to NR3C1 protein binds to HNF1A protein binds to SLC4A2 promoter alternative form]
Ursodeoxycholic Acid	NR3C1	Homo sapiens	[Ursodeoxycholic Acid co-treated with Dexamethasone] promotes the reaction [HNF1A protein binds to NR3C1 protein binds to SLC4A2 promoter]
Ursodeoxycholic Acid	NR3C1	Homo sapiens	[Ursodeoxycholic Acid co-treated with Dexamethasone] promotes the reaction [HNF1B protein binds to NR3C1 protein binds to EP300 protein binds to SLC4A2 promoter

			alternative form]
Ursodeoxycholic Acid	NR3C1	Homo sapiens	[Ursodeoxycholic Acid co-treated with Dexamethasone] promotes the reaction [NR3C1 protein binds to SLC4A2 promoter alternative form]
Ursodeoxycholic Acid	NR3C1	Homo sapiens	[Ursodeoxycholic Acid co-treated with Dexamethasone] results in increased localization of NR3C1 protein
Ursodeoxycholic Acid	NR3C1	Homo sapiens	[Ursodeoxycholic Acid co-treated with NR3C1] results in decreased expression of HLA-DRA mRNA
Ursodeoxycholic Acid	NR3C1	Homo sapiens	Ursodeoxycholic Acid results in increased localization of NR3C1 protein
Valproic Acid	NR3C1	Homo sapiens	[Hydralazine co-treated with Valproic Acid] results in increased expression of NR3C1 mRNA
Valproic Acid	NR3C1	Homo sapiens	[NOG protein co-treated with Valproic Acid co-treated with (6-(4-(2-piperidin-1-ylethoxy)phenyl))-3-pyridin-4-ylpyrazolo(1,5-a)pyrimidine co-treated with 4-(5-benzo(1,3)dioxol-5-yl-4-pyridin-2-yl-1H-imidazol-2-yl)benzamide] results in increased expression of NR3C1 mRNA
Valproic Acid	NR3C1	Homo sapiens	Valproic Acid results in decreased methylation of NR3C1 gene
Valproic Acid	NR3C1	Homo sapiens	Valproic Acid results in increased expression of NR3C1 mRNA
Vitamin K 3	NR3C1	Homo sapiens	Vitamin K 3 affects the expression of NR3C1 mRNA
vorinostat	NR3C1	Homo sapiens	vorinostat results in increased expression of NR3C1 mRNA
Zidovudine	NR3C1	Homo sapiens	Zidovudine affects the expression of NR3C1 mRNA
Zinc Oxide	NR3C1	Homo sapiens	Zinc Oxide analog results in decreased expression of NR3C1 mRNA
1,10-phenanthroline	ACE	Homo sapiens	1,10-phenanthroline results in decreased expression of ACE mRNA
Air Pollutants, Occupational	ACE	Homo sapiens	Air Pollutants, Occupational results in decreased methylation of ACE gene
Air Pollutants, Occupational	ACE	Homo sapiens	Air Pollutants, Occupational results in increased expression of ACE mRNA
Antirheumatic Agents	ACE	Homo sapiens	Antirheumatic Agents results in increased expression of ACE mRNA
Arsenates	ACE	Homo sapiens	[Atrazine co-treated with Arsenates] results in increased expression of ACE mRNA
Ascorbic Acid	ACE	Homo sapiens	Ascorbic Acid inhibits the reaction [epigallocatechin gallate results in decreased

			activity of ACE protein]
Aspirin	ACE	Homo sapiens	ACE polymorphism affects the susceptibility to Aspirin
Atrazine	ACE	Homo sapiens	[Atrazine co-treated with Arsenates] results in increased expression of ACE mRNA
benazepril	ACE	Homo sapiens	ACE gene polymorphism affects the susceptibility to benazepril
benazepril	ACE	Homo sapiens	benazepril results in decreased activity of ACE protein
benzyloxycarbonylleucyl-leucyl-leucine aldehyde	ACE	Homo sapiens	[Butyrates co-treated with benzyloxycarbonylleucyl-leucyl-leucine aldehyde] affects the localization of ACE protein mutant form
benzyloxycarbonylleucyl-leucyl-leucine aldehyde	ACE	Homo sapiens	[Butyrates co-treated with benzyloxycarbonylleucyl-leucyl-leucine aldehyde] results in increased activity of ACE protein mutant form
bisindolylmaleimide I	ACE	Homo sapiens	bisindolylmaleimide I inhibits the reaction [VEGFA protein results in increased expression of ACE protein]
bisphenol A	ACE	Homo sapiens	bisphenol A results in increased expression of ACE mRNA
boric acid	ACE	Homo sapiens	boric acid inhibits the reaction [epigallocatechin gallate results in decreased activity of ACE protein]
Bortezomib	ACE	Homo sapiens	[Butyrates co-treated with Bortezomib] results in increased activity of ACE protein mutant form
Butyrates	ACE	Homo sapiens	[Butyrates co-treated with benzyloxycarbonylleucyl-leucyl-leucine aldehyde] affects the localization of ACE protein mutant form
Butyrates	ACE	Homo sapiens	[Butyrates co-treated with benzyloxycarbonylleucyl-leucyl-leucine aldehyde] results in increased activity of ACE protein mutant form
Butyrates	ACE	Homo sapiens	[Butyrates co-treated with Bortezomib] results in increased activity of ACE protein mutant form
carvedilol	ACE	Homo sapiens	carvedilol inhibits the reaction [Tetradecanoylphorbol Acetate results in increased expression of ACE mRNA]
Chelating Agents	ACE	Homo sapiens	Chelating Agents analog results in decreased activity of ACE protein
Cilazapril	ACE	Homo sapiens	ACE gene polymorphism affects the susceptibility to Cilazapril
Cocaine	ACE	Homo	ACE inhibits the reaction [Cocaine affects the

		sapiens	abundance of Dinoprostone]
Cocaine	ACE	Homo sapiens	ACE inhibits the reaction [Cocaine affects the abundance of Epoprostenol]
Crack Cocaine	ACE	Homo sapiens	Crack Cocaine results in increased activity of ACE protein
Edetic Acid	ACE	Homo sapiens	Edetic Acid results in decreased activity of ACE protein
Edetic Acid	ACE	Homo sapiens	Zinc Sulfate inhibits the reaction [Edetic Acid results in decreased activity of ACE protein]
Enalapril	ACE	Homo sapiens	ACE gene polymorphism affects the susceptibility to [Enalapril co-treated with Sodium Chloride]
Enalapril	ACE	Homo sapiens	ACE gene polymorphism inhibits the reaction [[Enalapril co-treated with Sodium Chloride] results in increased susceptibility to AGT protein modified form]
Enalapril	ACE	Homo sapiens	Enalapril results in decreased activity of ACE protein
Enalaprilat	ACE	Homo sapiens	Enalaprilat inhibits the reaction [Glucose results in increased expression of ACE protein]
epigallocatechin gallate	ACE	Homo sapiens	Ascorbic Acid inhibits the reaction [epigallocatechin gallate results in decreased activity of ACE protein]
epigallocatechin gallate	ACE	Homo sapiens	boric acid inhibits the reaction [epigallocatechin gallate results in decreased activity of ACE protein]
epigallocatechin gallate	ACE	Homo sapiens	epigallocatechin gallate binds to and results in decreased activity of ACE protein
Estrogens, Conjugated (USP)	ACE	Homo sapiens	Estrogens, Conjugated (USP) results in decreased activity of ACE protein
ethylbenzene	ACE	Homo sapiens	[Toluene co-treated with ethylbenzene co-treated with Xylenes] results in decreased methylation of ACE gene
ethylbenzene	ACE	Homo sapiens	[Toluene co-treated with ethylbenzene co-treated with Xylenes] results in increased expression of ACE mRNA
Glucose	ACE	Homo sapiens	Enalaprilat inhibits the reaction [Glucose results in increased expression of ACE protein]
Glucose	ACE	Homo sapiens	Glucose results in increased expression of ACE protein
herbimycin	ACE	Homo sapiens	herbimycin inhibits the reaction [VEGFA protein results in increased expression of ACE protein]
Hydrogen Peroxide	ACE	Homo sapiens	[GPX1 gene polymorphism co-treated with ACE gene polymorphism] affects the susceptibility to Hydrogen Peroxide
ICG 001	ACE	Homo	ICG 001 inhibits the reaction [AGT protein

		sapiens	results in increased expression of ACE protein]
idrapril	ACE	Homo sapiens	idrapril results in decreased activity of ACE protein
Indomethacin	ACE	Homo sapiens	Indomethacin promotes the reaction [VEGFA protein results in increased expression of ACE protein]
Lisinopril	ACE	Homo sapiens	Lisinopril analog results in decreased activity of ACE protein
Losartan	ACE	Homo sapiens	Losartan inhibits the reaction [AGT protein results in increased expression of ACE protein]
Metoprolol	ACE	Homo sapiens	ACE gene polymorphism affects the susceptibility to Metoprolol
Mustard Gas	ACE	Homo sapiens	ACE gene affects the susceptibility to Mustard Gas
N-(2-cyclohexyloxy-4-nitrophenyl)methanesulfonamide	ACE	Homo sapiens	N-(2-cyclohexyloxy-4-nitrophenyl)methanesulfonamide promotes the reaction [VEGFA protein results in increased expression of ACE protein]
Nicotine	ACE	Homo sapiens	Nicotine results in increased expression of ACE mRNA
omapatrilat	ACE	Homo sapiens	omapatrilat results in decreased activity of ACE protein
Perindopril	ACE	Homo sapiens	ACE gene polymorphism affects the susceptibility to Perindopril
Dinoprostone	ACE	Homo sapiens	ACE inhibits the reaction [Cocaine affects the abundance of Dinoprostone]
Phenylephrine	ACE	Homo sapiens	ACE protein affects the susceptibility to Phenylephrine
Epoprostenol	ACE	Homo sapiens	ACE inhibits the reaction [Cocaine affects the abundance of Epoprostenol]
quinapril	ACE	Homo sapiens	quinapril results in decreased activity of ACE protein
Raloxifene Hydrochloride	ACE	Homo sapiens	Raloxifene Hydrochloride results in decreased expression of ACE mRNA
Ramipril	ACE	Homo sapiens	Ramipril results in decreased activity of ACE protein
sodium arsenite	ACE	Homo sapiens	ERN1 affects the reaction [sodium arsenite results in increased expression of ACE mRNA]
sodium arsenite	ACE	Homo sapiens	HIF1A affects the reaction [sodium arsenite results in increased expression of ACE mRNA]
sodium arsenite	ACE	Homo sapiens	sodium arsenite promotes the reaction [[XBP1 protein binds to HIF1A protein] which binds to ACE enhancer]
sodium arsenite	ACE	Homo sapiens	sodium arsenite results in increased expression of ACE mRNA
sodium arsenite	ACE	Homo sapiens	sodium arsenite results in increased expression of ACE protein

sodium arsenite	ACE	Homo sapiens	XBP1 affects the reaction [sodium arsenite results in increased expression of ACE mRNA]
Sodium Chloride	ACE	Homo sapiens	ACE gene polymorphism affects the susceptibility to [Enalapril co-treated with Sodium Chloride]
Sodium Chloride	ACE	Homo sapiens	ACE gene polymorphism inhibits the reaction [[Enalapril co-treated with Sodium Chloride] results in increased susceptibility to AGT protein modified form]
Tetradecanoylphorbol Acetate	ACE	Homo sapiens	carvedilol inhibits the reaction [Tetradecanoylphorbol Acetate results in increased expression of ACE mRNA]
Tetradecanoylphorbol Acetate	ACE	Homo sapiens	Tetradecanoylphorbol Acetate inhibits the reaction [VEGFA protein results in increased expression of ACE protein]
Tetradecanoylphorbol Acetate	ACE	Homo sapiens	Tetradecanoylphorbol Acetate results in increased expression of ACE mRNA
Toluene	ACE	Homo sapiens	[Toluene co-treated with ethylbenzene co-treated with Xylenes] results in decreased methylation of ACE gene
Toluene	ACE	Homo sapiens	[Toluene co-treated with ethylbenzene co-treated with Xylenes] results in increased expression of ACE mRNA
Valproic Acid	ACE	Homo sapiens	Valproic Acid results in increased methylation of ACE gene
Xylenes	ACE	Homo sapiens	[Toluene co-treated with ethylbenzene co-treated with Xylenes] results in decreased methylation of ACE gene
Xylenes	ACE	Homo sapiens	[Toluene co-treated with ethylbenzene co-treated with Xylenes] results in increased expression of ACE mRNA
zaprinast	ACE	Homo sapiens	zaprinast promotes the reaction [VEGFA protein results in increased expression of ACE protein]
Zinc	ACE	Homo sapiens	Zinc results in decreased activity of ACE protein
Zinc Sulfate	ACE	Homo sapiens	Zinc Sulfate inhibits the reaction [Edetic Acid results in decreased activity of A

Supplementary Appendix- Glossary of Terminology

Holistic approaches - analysis that takes into account the whole members of the problem including, molecular, physiological, and social factors in consideration.

Ethnobotanical- is the study of a region's plants and their practical uses through the traditional knowledge of a local culture and people

Interactome – the whole set of molecular interactions in a particular cell, the term specifically refers to physical interactions among molecules such as those among proteins, or between small molecules and proteins but can also describe sets of indirect interactions among genes.

Protein-protein interactions (PPI)- are the physical contacts of high specificity established between two or more protein molecules as a result of biochemical events steered by electrostatic forces including the hydrophobic effect.

Hubs- a hub is a node with a number of links that greatly exceeds the average

Bottlenecks- nodes with a high betweenness centrality

Clustering analysis- is the task of grouping a set of objects in such a way that objects in the same group (called a cluster) are more similar (in some sense) to each other than to those in other groups (clusters).

Druggability- is a term used in drug discovery to describe a biological target (such as a protein) that is known to or is predicted to bind with high affinity to a drug.

Tractability- The assessment of target tractability allows you to exploit target details, such as whether there is a binding site in the protein that can be used for small molecule binding, or an accessible epitope for antibody based therapy.

Proteostasis- is the concept that there are competing and integrated biological pathways within cells that control the biogenesis, folding, trafficking and degradation of proteins present within and outside the cell

DNA methylation- is a process by which methyl groups are added to the DNA molecule.

Epigenetic clock- is a term used to describe a biochemical test used to measure age, the test is based on DNA methylation levels.

Non-coding RNA molecules- is an RNA molecule that is not translated into a protein.

Immunosenescence- refers to the gradual deterioration of the immune system brought on by natural age advancement.

Comparative Toxicogenomics Database-

KEGG- Kyoto Encyclopedia of Genes and Genomes, is a collection of databases dealing with genomes, biological pathways, diseases, drugs, and chemical substances.

Reactome- is pathway database which provides intuitive bioinformatics tools for the visualisation, interpretation and analysis of pathway knowledge.

Sociomatrix- is a term used in social network analysis to arrange network information.

Degree- of a graph is the number of edges incident to the vertex

Closeness - of a node is a measure of centrality in a network, calculated as the reciprocal of the sum of the length of the shortest paths between the node and all other nodes in the graph.

Betweenness- is a measure of centrality in a graph based on shortest paths

Lipinski's Rule of five- rule describes molecular properties important for a drug's pharmacokinetics in the human body, including their absorption, distribution, metabolism, and excretion ("ADME"). However, the rule does not predict if a compound is pharmacologically active.

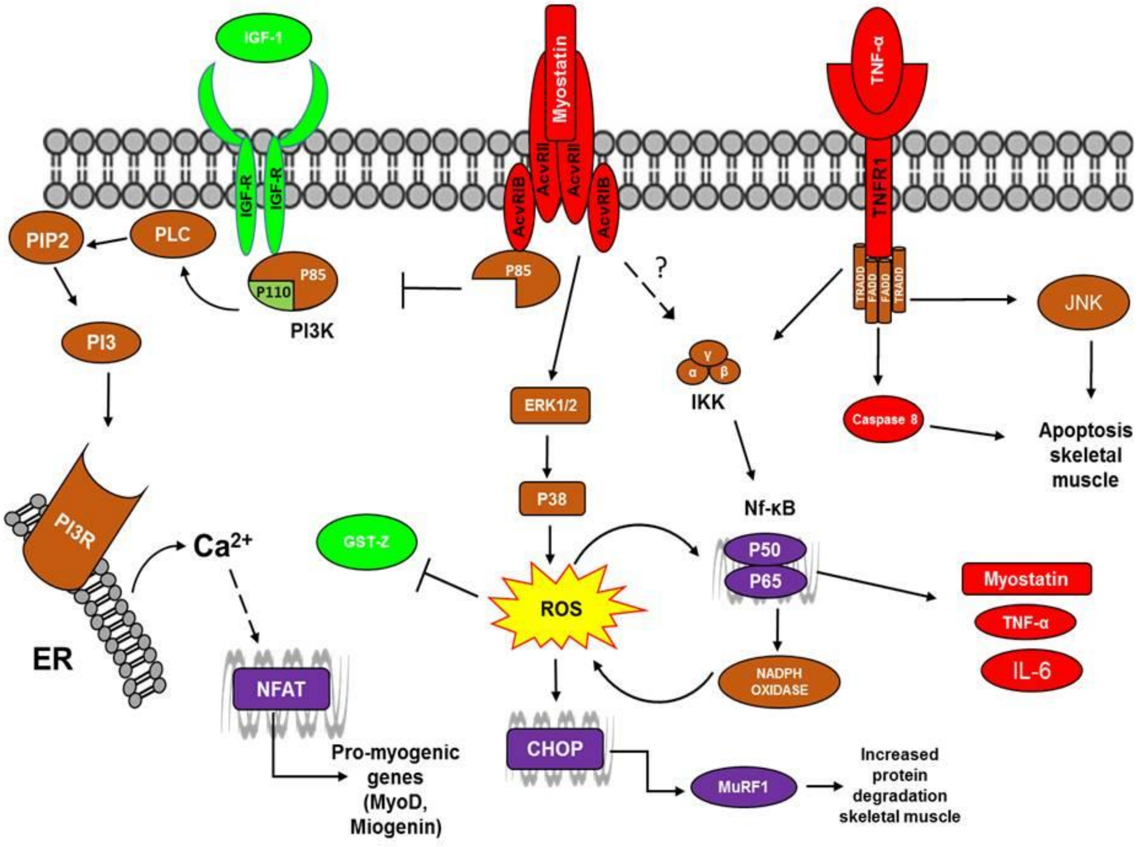

Supplementary Figure 1S.Proposed mechanism from Clusters 1 and 3. Clusters from the proteins related to frailty, musculoskeletal diseases, and apoptosis and with the proliferation and differentiation of the muscle tissue.

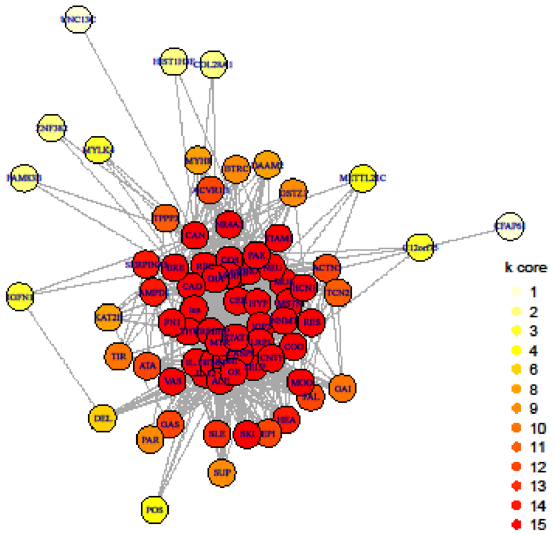

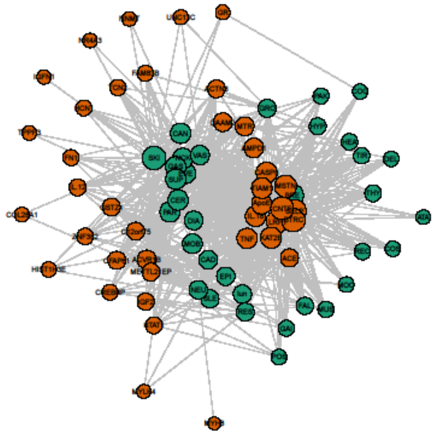

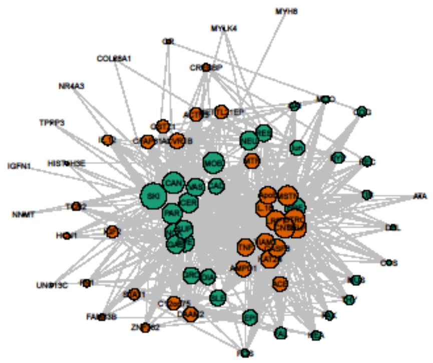

B) Node Size Closeness

C)Betweeneness

Supplementary Figure 2S. Deficits and genes network analysis: A) k-cores, B) Node size represents closeness, C) Node size represents betweenness. In B) and C), genes in orange and genes in green

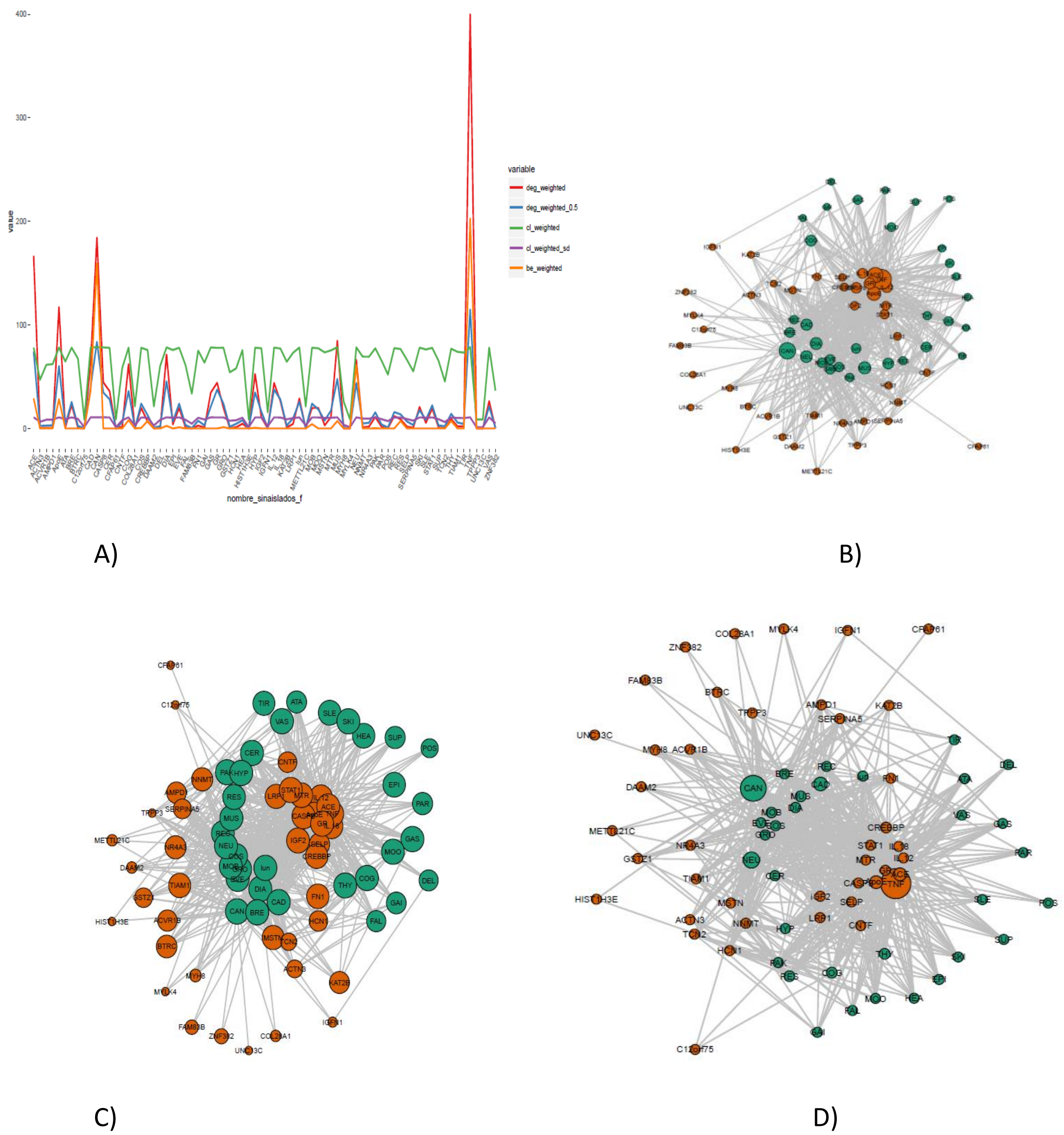

Supplementary Figure 3S. Network whose nodes correspond to deficits and genes considering weighted edges, and associated properties. A) Weighted centrality measures associated with each node; weighted degree without tuning parameter (red) and with a tuning parameter of 0.5 (blue), this considers both the number of connections and the weights; non-standardized weighted closeness(green) and standardized weighted closeness (purple); and weighted betweenness (orange). B) Network of deficits (green) and genes (orange), node size representing weighted degree with a tuning parameter of 0.5. C) Network of deficits (green) and genes (orange), node size representing standardized weighted closeness. D) Network of deficits (green) and genes (orange), node size representing weighted betweenness.
